# Post‐marketing safety of CGRP monoclonal antibodies and gepants: A systematic review of spontaneous reporting system data

**DOI:** 10.1111/head.70081

**Published:** 2026-03-15

**Authors:** Martina Giacon, Salvatore Terrazzino

**Affiliations:** ^1^ Department of Pharmaceutical Sciences University of Piemonte Orientale Novara Italy

**Keywords:** CGRP inhibitors, drug safety, Gepants, monoclonal antibodies, pharmacovigilance, systematic review

## Abstract

**Objective:**

Calcitonin gene‐related peptide (CGRP) inhibitors, including monoclonal antibodies (mAbs) and small‐molecule antagonists (gepants), have transformed migraine treatment. Although clinical trials established their efficacy and initial safety, post‐marketing surveillance is essential for understanding their real‐world safety profile in a broader population. Therefore, this study aims to systematically review and synthesize findings from published pharmacovigilance studies that analyze potential safety signals for CGRP inhibitors using major international databases, including the United States Food and Drug Administration (FDA) Adverse Event Reporting System (FAERS), the World Health Organization's (WHO's) VigiBase, and EudraVigilance, in order to establish a comprehensive real‐world safety profile to guide clinical practice.

**Methods:**

A systematic search was conducted in major electronic databases for studies published up to September 2025. Study selection, data extraction, and quality assessment were performed by two independent researchers. We included original research articles analyzing FAERS, VigiBase, or EudraVigilance for AEs associated with erenumab, galcanezumab, fremanezumab, eptinezumab, rimegepant, ubrogepant, atogepant, or zavegepant. Data on key signals of disproportionate reporting (SDRs) and quantitative measures of disproportionality were extracted and synthesized thematically.

**Results:**

The search identified 30 eligible studies. For mAbs, consistent SDRs included injection site reactions, alopecia (e.g., fremanezumab reporting odds ratio (ROR) ranging from 2.73 to 6.9), constipation (primarily for erenumab, RORs ranging from 4.92 to 17.94), and a range of cardiovascular events. For gepants, common SDRs included nausea and fatigue or somnolence, with highly specific SDRs for severe constipation for atogepant (ROR_025_ = 19.99) and dysgeusia for zavegepant (ROR_025_ = 212.07), linked to its nasal administration. A critical divergence was observed for rare but serious cerebrovascular events: SDRs for reversible cerebral vasoconstriction syndrome (RCVS [erenumab ROR 9.43, 95% confidence interval {CI} 4.5–19.8]) and cervical artery dissection (CeAD [galcanezumab ROR 14.0, 95% CI 6.22–31.4]) were associated with certain mAbs. Conversely, no such SDRs have been detected for gepants to date, although this distinction requires confirmation as real‐world exposure increases. However, a class‐level SDR for cerebrovascular diseases as a whole was identified for CGRP inhibitors as a group (ROR 1.22, 95% CI 1.12–1.33). Also notable were shared SDRs for Raynaud's phenomenon and alopecia across both subclasses. Finally, concerning safety in pregnancy, the data are complex: while comprehensive class‐level analyses did not identify a disproportionality signal compared to triptans, some analyses of individual drugs have identified reporting patterns that warrant cautious interpretation, underscoring the need for further dedicated pharmacovigilance studies to fully clarify the safety profile in this population.

**Conclusions:**

This systematic review confirms that CGRP inhibitors have a manageable yet complex safety profile. It distinguishes rare, serious cerebrovascular events (RCVS, CeAD) associated with some mAbs but not with gepants, as well as shared adverse effects such as Raynaud's phenomenon and alopecia. Significant heterogeneity in safety profiles—from erenumab's pronounced constipation SDR to zavegepant's unique dysgeusia—challenges the view of CGRP inhibitors as a monolithic category. These findings provide a clear rationale for personalized risk assessment, enabling clinicians to tailor treatment to individual patient profiles.

## Plain Language Summary

Calcitonin gene‐related peptide (CGRP) inhibitors are a newer class of migraine drugs that have proven effective and are now widely available, but how they behave in real‐world patients, and whether their side effects match what was seen in clinical trials, is still being understood. To address this, we combined findings from 30 safety studies that analyzed large international databases tracking drug side effects and found that while these drugs are generally well tolerated, each has its own set of risks and safety profiles; for instance, monoclonal antibodies were associated with constipation and injection site reactions, whereas oral gepants were linked to nausea and specific taste disturbances. Our findings highlight important differences, such as rare vascular events associated with some antibodies but not gepants, that enable clinicians to personalize migraine treatment and minimize risks for individual patients.

**Abbreviations**
AE: adverse event
AESI: adverse event of special interest
AOC: system organ class
CeAD: cerebral and cervical artery dissection
CGRP: calcitonin gene‐related peptide
CI: confidence interval
CNS: central nervous system
EEA: European Economic Area
EudraVigilance: European Union Drug Regulating Authorities Pharmacovigilance
FAERS: FDA Adverse Event Reporting System
FDA: Food and Drug Administration (United States)
IC: information component
ICSR: Individual Case Safety Report
INPLASY: International Platform of Registered Systematic Review and Meta‐analysis Protocols
IRR: infusion‐related reaction
mAbs: monoclonal antibodies
NA: not applicable
PRISMA: Preferred Reporting Items for Systematic Reviews and Meta‐Analyses
PRR: proportional reporting ratio
PT: Preferred Term
RCTs: randomized controlled trials
RCVS: reversible cerebral vasoconstriction syndrome
RECORD‐PE: REporting of studies Conducted using Observational Routinely collected health Data for Pharmacoepidemiology
ROR: reporting odds ratio
SDR: signal of disproportionate reporting
SRS: Spontaneous Reporting System
STROBE: STrengthening the Reporting of OBservational studies in Epidemiology
WHO: World Health Organization

## INTRODUCTION

Migraine is a common and disabling neurological disorder that is considered one of the leading causes of disability worldwide, especially in young adults and women.^1^ The burden goes beyond the headache itself and significantly affects quality of life, productivity, and mental health.^2^ The pathophysiology of migraine is complex, but the neuropeptide calcitonin gene‐related peptide (CGRP) has been identified as a key player in mediating nociceptive signaling and vasodilation associated with migraine attacks.^3^ This discovery has led to the development of a revolutionary class of targeted therapies: CGRP inhibitors.^4^ Historically, migraine prevention relied on non‐specific oral medications such as beta‐blockers, antiseizure medications, and antidepressants. However, due to limited efficacy and poor tolerability associated with these first‐line treatments,^5^ international guidelines generally recommend CGRP inhibitors for patients who have failed to respond to or are intolerant to standard oral preventive therapies, marking a significant shift in clinical management.^6^ This therapeutic class consists of two distinct categories of drugs that target the CGRP signaling pathway. The first category includes long‐acting monoclonal antibodies (mAbs) administered by injection or infusion for prophylactic treatment.^7^ These therapies target either the CGRP ligand itself (galcanezumab, fremanezumab, and eptinezumab) or the blockade of its receptor (erenumab). The second category consists of small molecule CGRP receptor antagonists, known as gepants, which are administered orally or nasally.^8^ This versatile class includes options for acute treatment (ubrogepant and zavegepant), preventive treatment (atogepant), and both acute and preventive therapy (rimegepant).

The introduction of these new therapeutic classes requires a rigorous evaluation of their safety profile. While pre‐approval randomized controlled trials (RCTs) are the gold standard for demonstrating efficacy and a basic safety profile, their inherent limitations‐ such as strict inclusion/exclusion criteria, relatively small sample sizes and limited follow‐up duration‐ prevent a comprehensive assessment of rare, delayed, or long‐term adverse events (AEs).^9^ Indeed, even a large‐scale synthesis of these trials, a network meta‐analysis based on 19 clinical trials and 14,584 patients,^10^ has confirmed that CGRP inhibitors are a safe option for migraine prevention. In particular, no significant differences in serious AEs were found between the drugs and placebo. However, the analysis distinguished the tolerability profiles of each drug and found that atogepant and galcanezumab were associated with a higher incidence of treatment‐related AEs compared to other drugs in the same class, such as eptinezumab.^10^ Nevertheless, real‐world populations are far more heterogeneous and often include patients with multiple comorbidities and concomitant medications that are usually excluded from RCTs. Therefore, post‐marketing pharmacovigilance is an important and necessary part of the drug safety lifecycle.^11^ Large spontaneous reporting system (SRS) databases, most notably the U.S. FAERS and WHO's VigiBase, serve as important tools for this surveillance.^12^, ^13^ Specifically, the U.S. FDA Adverse Event Reporting System (FAERS) aggregates reports submitted by healthcare professionals, consumers, and manufacturers, predominantly from the United States. The World Health Organization's (WHO's) VigiBase, managed by the Uppsala Monitoring Centre, stands as the largest global database, pooling anonymized reports from national pharmacovigilance centers in over 130 member countries. Similarly, EudraVigilance acts as the centralized European database for managing and analyzing reports of suspected adverse reactions from the European Economic Area (EEA). As these databases collect millions of Individual Case Safety Reports (ICSRs) worldwide, researchers can use disproportionality analysis to detect signals of disproportionate reporting (SDRs), which may indicate potential safety signals, such as a notable incidence of constipation, particularly with erenumab,^14^ and rare cerebrovascular events such as reversible cerebral vasoconstriction syndrome (RCVS),^15^ which may not have been apparent during clinical development.

Given the widespread and increasing use of CGRP inhibitors and the fact that a critical mass of pharmacovigilance studies is now available, a comprehensive synthesis of their real‐world safety data is essential. While individual pharmacovigilance studies are crucial for the detection of initial signals of disproportionate reporting, they alone cannot provide a definitive overview of the safety profile of a drug class. Each study is limited by its specific design‐ the choice of database (which can lead to geographical bias), the time period analyzed, and the statistical methods used. A potential safety signal only gains credibility and moves from a mere observation to a robust result if it is consistently identified in multiple analyzes using different methodological approaches. While data sources often overlap (as VigiBase aggregates national reports), the principle of consistent replication across different study designs is crucial for distinguishing a genuine safety issue from a statistical anomaly or reporting bias. Therefore, this systematic review aims to summarize and synthesize the evidence on the real‐world safety profiles of CGRP inhibitors from the entire landscape of published pharmacovigilance studies. This approach provides a more thorough and up‐to‐date overview of the safety profiles of both the monoclonal antibody and gepant classes of CGRP inhibitors and offers a perspective beyond the scope of a single paper. Such a comprehensive overview is crucial to inform clinical decisions, guide future research, and support regulatory assessments.

## METHODS

### Search strategy and study selection

The protocol was defined in advance of the literature search and registered on the International Platform of Registered Systematic Review and Meta‐analysis Protocols (INPLASY) (registration number 202560067. doi:10.37766/inplasy2025.9.0025). This systematic review was conducted in accordance with the Preferred Reporting Items for Systematic Reviews and Meta‐Analyses (PRISMA) 2020 statement.^16^ A comprehensive literature search was conducted in four electronic databases (PubMed, Web of Science, Cochrane Library, and OpenGrey via the DANS repository mirror: https://lifesciences.datastations.nl/) up to September 22, 2025. To ensure broad sensitivity and consistency across platforms, a keyword‐based Boolean search strategy was applied to the Title/Abstract or Topic fields of each database. The full search strings and parameters for each database are detailed in Table S1.

(FAERS OR VigiBase OR EudraVigilance OR Pharmacovigilance OR adverse event reporting OR spontaneous reporting) AND (CGRP inhibitor OR CGRP inhibitors OR CGRP antagonist OR CGRP antagonists OR anti‐CGRP monoclonal antibody OR anti‐CGRP monoclonal antibodies OR gepant OR gepants OR Erenumab OR Aimovig OR Galcanezumab OR Emgality OR Fremanezumab OR Ajovy OR Eptinezumab OR Vyepti OR Rimegepant OR Nurtec OR Vydura OR Ubrogepant OR Ubrelvy OR Atogepant OR Qulipta OR Zavegepant OR Zavzpret).

Two researchers (M.G. and S.T.) independently reviewed all articles. Discrepancies regarding inclusion or exclusion were resolved through consensus discussions to reach 100% agreement; inter‐rater reliability statistics were not retrospectively calculated. Original research articles that performed a pharmacovigilance analysis of one or more CGRP inhibitors using FAERS, VigiBase, or EudraVigilance data were considered. Non‐research articles, such as editorials, letters, commentaries, and conference abstracts, were excluded. The scope of the review was deliberately limited to the class of CGRP inhibitors to provide a comprehensive assessment of therapies targeting this specific pathway. Although mAbs and gepants differ significantly in formulation, pharmacokinetics, and route of administration, evaluating them together allows for the discrimination between mechanism‐based class effects (resulting from CGRP pathway inhibition) and molecule‐specific effects (related to structure or metabolism). This focused approach allows for a cohesive analysis of mechanism‐based AEs and facilitates a meaningful comparison between the two subclasses. Conversely, the inclusion of older migraine drugs as primary subjects would have led to considerable methodological heterogeneity given their different mechanisms of action and well‐established, decades‐old safety profiles. Therefore, older therapies were not the focus of this review, although they were considered when used as active comparators within the included studies. It would also have diluted the focus on the emerging real‐world evidence for this new class of therapies.

### Data extraction and synthesis

To ensure consistency, two independent researchers (M.G. and S.T.) extracted data from the included studies using a standardized template. Any inconsistencies were resolved through iterative consensus discussions until 100% agreement was reached on the final dataset; therefore, a formal inter‐rater reliability statistic (e.g., Cohen's Kappa) was not calculated. Authors of the primary studies were not contacted to obtain additional data or clarification; the analysis relied solely on the information available in the published articles. For each study, we extracted key information on study characteristics (author, year, databases, time period), population data (number of reports, demographics), and key outcomes (SDRs and the specific disproportionality analyzes used, such as reporting odds ratio (ROR), proportional reporting ratio (PRR), or information component (IC)). We then performed a thematic synthesis to summarize the results. To highlight consistent SDRs, we organized the results by drug class (mAbs and gepants) and specific AEs of special interest (AESIs). Consistent with the known physiological mechanisms of CGRP, we focused the synthesis on pre‐defined key domains mapped to relevant system organ classes (SOCs), specifically Gastrointestinal, Cardiovascular, and Nervous System disorders, and included quantitative measures of disproportionality where reported. Review management, data extraction, and the calculation of descriptive statistics for study characteristics were performed using Microsoft Excel (Microsoft Corporation, Redmond, WA, USA). The range plots visualizing the consistency of safety signals (Figures 2 and 3) were generated using the Python programming language (version 3.12.12), utilizing the Pandas (v2.2.2), Seaborn (v0.13.2), and Matplotlib (v3.10.0) libraries. A quantitative meta‐analysis was not performed due to the substantial heterogeneity across studies regarding statistical methods (e.g., frequentist vs. Bayesian algorithms), comparator groups, and data sources.

### Methodological quality assessment

Two reviewers (M.G. and S.T.) independently assessed the methodological quality of each study using a predefined 12‐point checklist. Discrepancies were discussed until 100% agreement was reached on the final score for each item; a formal inter‐rater reliability statistic was not calculated. The predefined 12‐point checklist was specifically adapted for pharmacovigilance studies analyzing SRSs such as FAERS and was based on the core principles of the STrengthening the Reporting of OBservational studies in Epidemiology (STROBE)^17^ and RECORD‐PE guidelines.^18^ The checklist assessed five key areas: (1) clarity of objectives, (2) rigor of data management, (3) appropriateness of statistical analysis, (4) handling of bias, and (5) prudence of interpretation. Each of the 12 items was rated on a three‐point scale (Yes = 2, Partly = 1, No = 0). A final quality rating (High, Moderate, Low, or Critically Low) was then assigned based on the overall score and, crucially, the fulfillment of four main criteria essential to the validity of the study. These criteria were: handling of duplicates, appropriate statistical analysis, recognition of study limitations, and avoidance of causal formulations. The full checklist and detailed assessment criteria can be found in Table S2.

## RESULTS

### Literature search and study selection process

**FIGURE 1:**
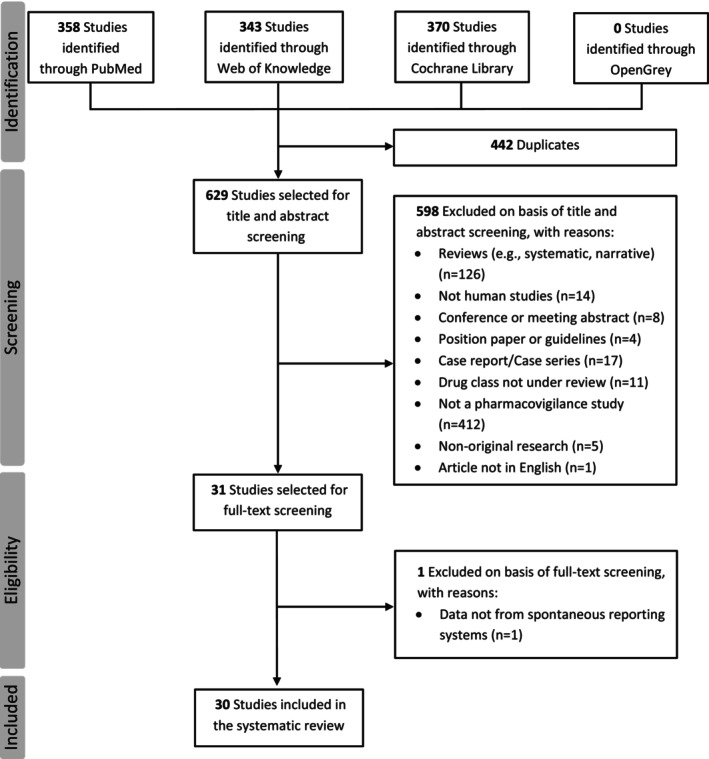
PRISMA flow diagram of the study selection process. The diagram illustrates the flow of information through the different phases of the review, including the number of records identified through database searching, records screened after duplicate removal, full‐text articles assessed for eligibility, and the final number of studies included in this systematic review. PRISMA, Preferred Reporting Items for Systematic Reviews and Meta‐Analyses.

The systematic literature search across four electronic databases (PubMed, Web of Knowledge, Cochrane Library, and OpenGrey) yielded a total of 1071 records. After removing 442 duplicates, 629 unique studies remained for title and abstract screening. During the title and abstract screening phase, 598 records were excluded for not meeting the predefined inclusion criteria. The primary reasons for exclusion at this stage were that the studies were not pharmacovigilance analyses (*n* = 412) or were systematic or narrative reviews (*n* = 126). Other reasons for exclusion included case reports or case series (*n* = 17), non‐human studies (*n* = 14), studies on an incorrect drug class (*n* = 11), conference abstracts (*n* = 8), non‐original research (*n* = 5), position papers or guidelines (n = 4), and one article not published in English (n = 1). The remaining 31 studies advanced to full‐text assessment for eligibility. Upon detailed review, one study was excluded because it did not utilize data from SRSs. Consequently, a final total of 30 studies met all inclusion criteria and were included in the systematic review (Figure 1).^14^, ^15^, ^19^, ^20^, ^21^, ^22^, ^23^, ^24^, ^25^, ^26^, ^27^, ^28^, ^29^, ^30^, ^31^, ^32^, ^33^, ^34^, ^35^, ^36^, ^37^, ^38^, ^39^, ^40^, ^41^, ^42^, ^43^, ^44^, ^45^, ^46^

### Characteristics of included studies

The characteristics of the 30 included retrospective pharmacovigilance analyses, all published between 2021 and 2025, are summarized in Table 1. The publication timeline indicates a rapidly expanding research field, with a peak in 2024–2025 (Figure S1A). Most analyses used data from the FAERS, which was the source for 20 studies, followed by the WHO's VigiBase® and European EudraVigilance (Figure S1B). Despite varied study scopes, a consistent demographic profile emerged, with predominantly female patients (>70%) aged 18–64 years. The research demonstrates strong international engagement, led by authors in China, the United States, and Europe (Figure S1C). The distribution of research reflects market introduction timelines, with more studies focused on mAbs than on gepants (Figure S2A). Accordingly, the first‐approved mAbs (erenumab, galcanezumab, fremanezumab) and the gepant rimegepant were the most frequently investigated drugs (Figure S2B). Primary research endpoints were divided between assessments of the overall safety profile (*n* = 15) and investigations into AESIs. Cardiovascular safety, including Raynaud's phenomenon, was the most common AESI, followed by safety in pregnancy, alopecia, and tinnitus (Figure S2C).

**TABLE 1:** Pharmacovigilance studies of CGRP inhibitors included in the systematic review.

Author & year	Drugs analyzed	Database (time period)	Comparator group	Safety focus	Total drug‐specific reports, *N* ^a^	% Female and median or mean age	Key AEs / SDRs identified	Type of Analysis	Method. quality
Noseda et al.^19^	Erenumab, Galcanezumab, Fremanezumab	VigiBase (up to 31 December 2019)	All other drugs in VigiBase; Triptans	Safety in pregnancy and lactation	94 (Erenumab: 50; Galcanezumab: 31; Fremanezumab: 13)	97.9% female; median age: 28	Spontaneous abortion, maternal toxicities, premature birth, birth defects.	ROR	M
Saely et al.^20^	Erenumab	FAERS (May 2018–April 2020) and retrospective Case Series	None/Descriptive study	Hypertension	61 (cases of elevated BP)	86% female; median age: 56	Elevated blood pressure, hypertension	Case series analysis, drug‐event causal association assessment	H
Sessa and Andersen^21^	Erenumab	FAERS (Q1 2018–Q2 2020)	Other drugs used for acute or preventative treatment of migraine (triptans, antidepressants [mirtazapine, amitriptyline], antiseizure medications [topiramate], OnabotulinumtoxinA, other CGRP mAbs [fremanezumab, galcanezumab])	General safety profile	23,312	64.8% female; median age: 50	SDRs: alopecia, depression, anxiety, myocardial infarction, increased heart rate, pulmonary embolism, weight alteration, insomnia, tinnitus, influenza‐like symptoms. Frequent AEs: injection‐site reactions, constipation, muscle spasms.	ROR	H
Gérard et al.^22^	Erenumab, galcanezumab, fremanezumab, eptinezumab; ubrogepant, rimegepant, atogepant	VigiBase (up to 31 January 2022)	All other drugs in VigiBase; triptans; Beta‐blockers (atenolol, metoprolol, nadolol, propranolol, timolol)	RP	99 (RP cases involving CGRP‐targeting drugs)	92.5% female; median age: 45	RP	IC	L
Liang and Sessa^23^	Erenumab	Eudravigilance (October 2019–October 2020) (compared with data of Sessa et al. 2021 from FAERS)	Sumatriptan	General safety profile	3381	86.4% female; most prevalent age group was 18–64 years (53.9%).	Deep‐vein thrombosis, spontaneous abortion, ileus paralytic, fecaloma, intestinal obstruction, nasopharyngitis, pneumonia, increased liver function biomarkers, increased C‐reactive protein, antinuclear antibody increased	ROR	L
Woods^24^	Erenumab, galcanezumab, fremanezumab, eptinezumab, rimegepant, ubrogepant, atogepant	FAERS (Up to Q4 2021)	All other drugs in the database; triptans; celecoxib; anticonvulsants; onabotulinumtoxinA; beta‐blockers	Alopecia	56,251 (erenumab: 32,873; galcanezumab: 15,687; fremanezumab: 3745; rimegepant: 2352; eptinezumab: 900; ubrogepant: 674; atogepant: 20)	70.3% female; mean age: 46	Alopecia	PRR	L
Battini et al.^25^	Ubrogepant and rimegepant	FAERS (Up to Q3 2021)	Erenumab	General safety profile	2010 (ubrogepant), 3691 (rimegepant)	Ubrogepant: 85.9% female; median age 45 years. Rimegepant: 88.0% female; median age 53 years	Ubrogepant: visual hallucination, pallor, personality change, skin exfoliation, facial paralysis, epistaxis, sepsis, dysphagia, skin burning sensation, drug hypersensitivity. Rimegepant: petit mal epilepsy, blood glucose decreased, atrial fibrillation, emotional distress, panic reaction, oral discomfort, stomatitis, gastritis, oral paresthesia/hypoesthesia, hypersomnia, seizure	ROR	H
Noseda et al.^26^	Erenumab, galcanezumab, fremanezumab, eptinezumab	VigiBase® (up to 31 December 2021)	Entire database (all time); entire database (since 2018); triptans	Safety in pregnancy	286 (erenumab: 116; galcanezumab: 125; fremanezumab: 39; eptinezumab: 6)	95.5% female; median age: 33	Spontaneous abortion, maternal outcomes, fetal growth restriction, prematurity, neonatal outcomes	ROR	M
Ruiz et al.^27^	Erenumab, fremanezumab, galcanezumab, eptinezumab, rimegepant, ubrogepant, atogepant	FAERS (up to 18 August 2022) & Case Series	None/Descriptive study	Alopecia	1943 (erenumab: 1158; galcanezumab: 554; fremanezumab: 175; eptinezumab: 23; rimegepant: 26; ubrogepant: 4; atogepant: 3)	Predominantly female (ranging from 92.8% to 100% in FAERS reports); mean age 45.1–51.1 years	Alopecia	Case series and descriptive analysis of FAERS data	L
Silberstein et al.^28^	Erenumab, fremanezumab, galcanezumab	FAERS (for the first 6 months post‐launch for each drug between May 2018 and March 2019) (with IQVIA data for patient exposure estimates)	None (Descriptive analysis of reporting rates per 1000 exposed patients)	General safety profile during the first 6 months post‐launch	6908 (erenumab: 5468; fremanezumab: 450; galcanezumab: 990)	N/A	Migraine, headache, drug ineffective, injection‐site reactions (pain, erythema, swelling), constipation (erenumab), nausea	Reporting Rates (RR) per 1000 exposed patients	M
Cao et al.^29^	Ubrogepant	FAERS (Dec 2019 – Dec 2022)	All other drugs in the FAERS database	General safety profile	2067	67.4% female, predominantly aged 18 to 64 years	Migraine, nausea, somnolence, paraesthesia oral, dizziness, hemiparesis, mental impairment, dysstasia, tinnitus, chest pain, cold sweat, neck pain	ROR, PRR, IC, EBGM	M
Favrelière et al.^15^	Erenumab	VigiBase® (up to 31 May 2023)	All other drugs in VigiBase (excluding COVID‐19 vaccines)	RCVS	11	72.5% female, primarily in the 45–64 (40%) and 18–44 (35%) age groups	RCVS	ROR	L
Hu et al.^30^	Rimegepant	FAERS (Q2 2020 – Q1 2023)	All other drugs in the database	General safety profile	5806	86.6% female; mean age: 50.8 years	Vomiting projectile, eructation, motion sickness, feeling drunk, reaction to food additive, nausea, dyspepsia, abdominal discomfort	ROR, PRR, BCPNN, MGPS	H
Liang et al.^31^	Rimegepant, atogepant, ubrogepant	VigiAccess (up to 31 March 2024) & FAERS (Q1 2020–Q4 2023)	All other drugs in the database	Comparative safety profile on gastrointestinal, cardiovascular, hepatic, renal, and skin‐related AEs.	23,542 (rimegepant: 13,856; atogepant: 6208; ubrogepant: 3478)	Sixfold female‐to‐male ratio, predominant age group: 45–64	Nausea, constipation, dizziness, somnolence, fatigue, RP, alopecia, drug ineffective, migraine, headache	ROR	L
Noseda et al.^32^	Erenumab, galcanezumab, fremanezumab, eptinezumab, ubrogepant, rimegepant, atogepant	VigiBase® (up to 31 May 2023)	Triptans (sumatriptan, naratriptan, zolmitriptan, rizatriptan, almotriptan, eletriptan, frovatriptan)	Safety in pregnancy	467 (reports reporting exposure in pregnancy)	92% female; median age range: 31–34 years	No significant SDRs identified for overall pregnancy‐related reports, maternal outcomes, or fetal/neonatal outcomes compared to triptans.	ROR	M
Pan and Lin^33^	Rimegepant	FAERS (Q2 2020–Q1 2024)	All other drugs in the FAERS database	General safety profile	6659	59.9% female; the majority of cases (21.0%) were in the 45–65 age group	Nausea, medication overuse headache, somnolence, nasal edema, tension headache, performance status decreased, motion sickness, vertigo, hyperacusis, RP, panic attack, fear	ROR, PRR, IC, EBGM	M
Singh et al.^34^	galcanezumab, fremanezumab, erenumab, rimegepant	FAERS via OpenVigil2.1 (Q1 2004–Q3 2022)	All other drugs in the database	RP	86 (galcanezumab: 28; erenumab: 45; fremanezumab: 9; rimegepant: 4)	Majority of RP cases were in females and in the 18–64 age group	RP	ROR, PRR	L
Sorbara et al.^35^	Erenumab, galcanezumab, fremanezumab, eptinezumab	EudraVigilance (July 2018 – Dec 2022)	All other monoclonal antibodies in the database	Cardiovascular safety	9441 (erenumab: 5560; galcanezumab: 2020; fremanezumab: 1793; eptinezumab: 66)	82.6% female; 73.4% were in the 18–64 years age group	Hypertension, pallor, deep vein thrombosis, hot flush, palpitations, atrial fibrillation, myocardial infarction	ROR	H
Sun et al.^14^	Erenumab, galcanezumab, fremanezumab, eptinezumab	FAERS (Q1 2018 – Q1 2023)	All other drugs in FAERS	General safety profile	65,792 (erenumab: 38,515; galcanezumab: 19,485; fremanezumab: 5332; eptinezumab: 2460)	72.1% female; mean age: 48.7	Injection site reactions, constipation, alopecia, fatigue, throat irritation, pruritus, RP, weight increase, menstrual disorders, throat tightness, oral paraesthesia	ROR, BCPNN	H
Zhang et al.^36^	Atogepant	FAERS (Q3 2021–Q4 2023)	All other drugs in the database	General safety profile	3015	78.1% female, mean age: N/A	48 SDRs identified, including nausea, constipation, somnolence, decreased appetite, vertigo, migraine, headache, disturbance in attention, brain fog, RP, and cardiac flutter	ROR, PRR, BCPNN, MGPS	H
Chen et al.^37^	Eptinezumab	FAERS (Q2 2020 – Q3 2024), Meta‐analysis of 6 clinical trials (*n* = 3148).	All other drugs in the database	General safety profile	5306	62.6% female; 59.8% of cases in the 18–65 age group	Drug ineffective, migraine, therapeutic response shortened, throat irritation, drug effect less than expected, nasal congestion, hypersensitivity, anaphylactic reaction, increased intracranial pressure, facial paralysis	FAERS: ROR, PRR, MGPS, BCPNN; Meta‐analysis: RR	H
Cho et al.^38^	CGRP antagonists (class level)	VigiBase (1968–2024)	All other migraine medications in VigiBase	Cerebrovascular diseases	128,552 (for CGRP antagonists class)	71.0% female; most cases in the 45–64 age group	Cerebrovascular diseases	ROR, IC	L
Kim et al.^39^	fremanezumab, galcanezumab, eptinezumab, erenumab, rimegepant, ubrogepant, atogepant	FAERS (Q1 1968–Q3 2023)	All other drugs in the database; triptans.	Tinnitus	81,147 (erenumab: 41,117; galcanezumab: 20,390; rimegepant: 6493; fremanezumab: 5835; eptinezumab: 2862; atogepant: 2787; ubrogepant: 1663)	70.1% (CGRP inhibitors), 75.4% (triptans) female; age range: 21–77 (CGRP inhibitors), 15–81 (triptans)	Tinnitus	PRR, ROR	L
Lee et al.^40^	galcanezumab, fremanezumab, erenumab, eptinezumab, ubrogepant, rimegepant, atogepant	FAERS (from each drug's approval year through Q2 2023)	All other drugs in the database; active comparators (beta‐blockers, triptans, NSAIDs)	General safety profile	81,145	63.8–81.2% females; patients aged 18–65 years	Underdose, alopecia, and constipation, weight increased, somnolence dizziness, therapy interruption, injection site reactions, nausea	ROR, PRR, IC	H
Lee et al.^41^	Rimegepant, ubrogepant, atogepant, erenumab, galcanezumab, fremanezumab	FAERS (From approval year of each drug through August 2023)	All other drugs in the database; Other migraine therapies (triptans, beta‐blockers, onabotulinumtoxinA, anticonvulsants, NSAIDs)	RP	89 (cases of Raynaud's Phenomenon); total drug‐specific reports not explicitly reported.	Majority of patients female, aged 18–64 years	RP	ROR, IC	H
Nikitina et al.^42^	Eptinezumab, fremanezumab, galcanezumab, and erenumab.	EudraVigilance (from marketing authorization date up to 16 June 2024)	Topiramate	General safety profile	14,285 (eptinezumab: 501; erenumab: 7547; fremanezumab: 3241; galcanezumab: 2996)	83.6%–88.7% females; patients aged 18–64 years	Eptinezumab: ~40 SDRs including palpitations, oropharyngeal pain, erythema, anaphylactic reaction. Erenumab: ~40 SDRs including constipation, hypertension, muscle spasms, alopecia. Galcanezumab: ~50 SDRs including injection site reactions, RP, weight increased, constipation. Fremanezumab: ~30 SDRs including injection site swelling, pruritus, RP	ROR, IC, EBGM	M
Song et al.^43^	Rimegepant, atogepant, ubrogepant, zavegepant	FAERS (Jan 2020–Dec 2024)	All other drugs in the database	General safety profile	23,859 (rimegepant: 7766; atogepant: 3672; ubrogepant: 1958; zavegepant: 463)	Majority of patients female; age group 45–64 for rimegepant, atogepant, and ubrogepant, 18–44 for zavegepant	Rimegepant: feeling abnormal, nausea, somnolence, insomnia, anxiety, dysgeusia. Atogepant: Constipation, nausea, fatigue, alopecia, somnolence, insomnia. Ubrogepant: Fatigue, nausea, somnolence, dizziness, dry mouth. Zavegepant: Dysgeusia, taste disorder, nasal discomfort, ageusia, nausea	ROR, PRR, IC	H
Tokuyasu et al.^44^	Galcanezumab, fremanezumab, erenumab and eptinezumab, with a specific focus on galcanezumab	FAERS (Q1 2012 – Q4 2023) and a case report	All other drugs in the database; Sumatriptan (active comparator)	CeAD	69,906 total reports on CGRP mAbs (20,946 for galcanezumab), of these 10 were CeAD (6 for galcanezumab)	Demographic data for the full FAERS cohort was not provided. Case report patient: 39‐year‐old female	CeAD	ROR, Case Report analysis	H
Wen et al.^45^	Atogepant	FAERS (Q3 2021–Q3 2023)	All other drugs in the database (FAERS)	General safety profile	2876	78.6% female, predominant age group: 45–65	Constipation, nausea, fatigue, dizziness, somnolence, decreased appetite, migraine, tension headache, cluster headache, post‐concussion syndrome, abnormal dreams, self‐injurious ideation, brain fog, brain neoplasm, feeling abnormal, euphoric mood, hyperacusis	ROR, PRR, BCPNN, EBGM	H
Zheng et al.^46^	Erenumab, galcanezumab, fremanezumab, atogepant, rimegepant (among 562 total drugs analyzed)	FAERS (Q1 2004–Q2 2024)	All other drugs in the FAERS database	RP	4303 (total RP cases) *Breakdown by drug*: erenumab: 55; galcanezumab: 42; fremanezumab: 15; atogepant: 4; rimegepant: 8	Female predominance (ratio 3.1:1); cases concentrated in the 25–59 age group	RP	ROR, PRR, BCPNN, MGPS	H

Abbreviations: AE, adverse effects; BCPNN, Bayesian confidence propagation neural network; BP, blood pressure; CeAD, Cervical Artery Dissection; CV ICSRs, Cardiovascular Individual Case Safety Reports; EBGM, Empirical Bayes Geometric Mean; FAERS, FDA Adverse Event Reporting System; FDA, United States Food and Drug Administration; H, high; IC, Information Component; ICSF, Individual Case Safety Report; L, low; M, moderate; Method., methodological; MGPS, Multi‐item Gamma Poisson Shrinker; N/A, not provided in the study; NSAIDs, non‐steroidal anti‐inflammatory drugs; PRR, proportional reporting ratios; RCVS, reversible cerebral vasoconstriction syndrome; ROR, reporting odds ratio; RP, Raynaud's phenomenon; RR, risk ratio; SDR, signal of disproportionate reporting.

^a^ A Total Drug‐Specific Reports refers to the total number of individual case safety reports extracted for the specific drug(s) of interest in the study, serving as the denominator for that specific analysis.

### Methodological quality assessment

The methodological quality of the 30 included pharmacovigilance studies was assessed using a 12‐point checklist (Table S2). Based on the predefined criteria, 14 studies were rated as ‘High quality,’ seven as ‘Moderate quality,’ and nine as “Low quality” (Table 1). The total score ranged from 13 to 24. A score of 0 for any of the four main criteria (items 5, 6, 9, 11) resulted in a lower quality rating regardless of the overall score. The score for the handling of duplicate case reports (item 5), a main criterion, varied between studies (Table S3). Seventeen studies received a full score of 2, four studies received a partial score of 1, and nine studies received a score of 0. For the use of sensitivity or stratification analyses (item 8), 15 studies received a score of 2, three studies received a partial score of 1, and 12 studies received a score of 0. The low quality rating for nine studies was determined by the score of 0 on item 5, although the overall score was often high (e.g. 22/24). Three of the included studies were descriptive pharmacovigilance studies that did not perform disproportionality analysis.^20^, ^27^, ^28^ As these studies are descriptive and do not include the calculation of disproportionality metrics (such as ROR or PRR), the checklist items related to these statistical methods (items 6 and 7) were assessed as not applicable (NA). Consequently, the maximum possible quality score for these three studies was 20 out of the standard 24 points (Table S3).

### Focused narrative synthesis of adverse events

#### Post‐marketing safety profile of anti‐CGRP mAbs

**FIGURE 2:**
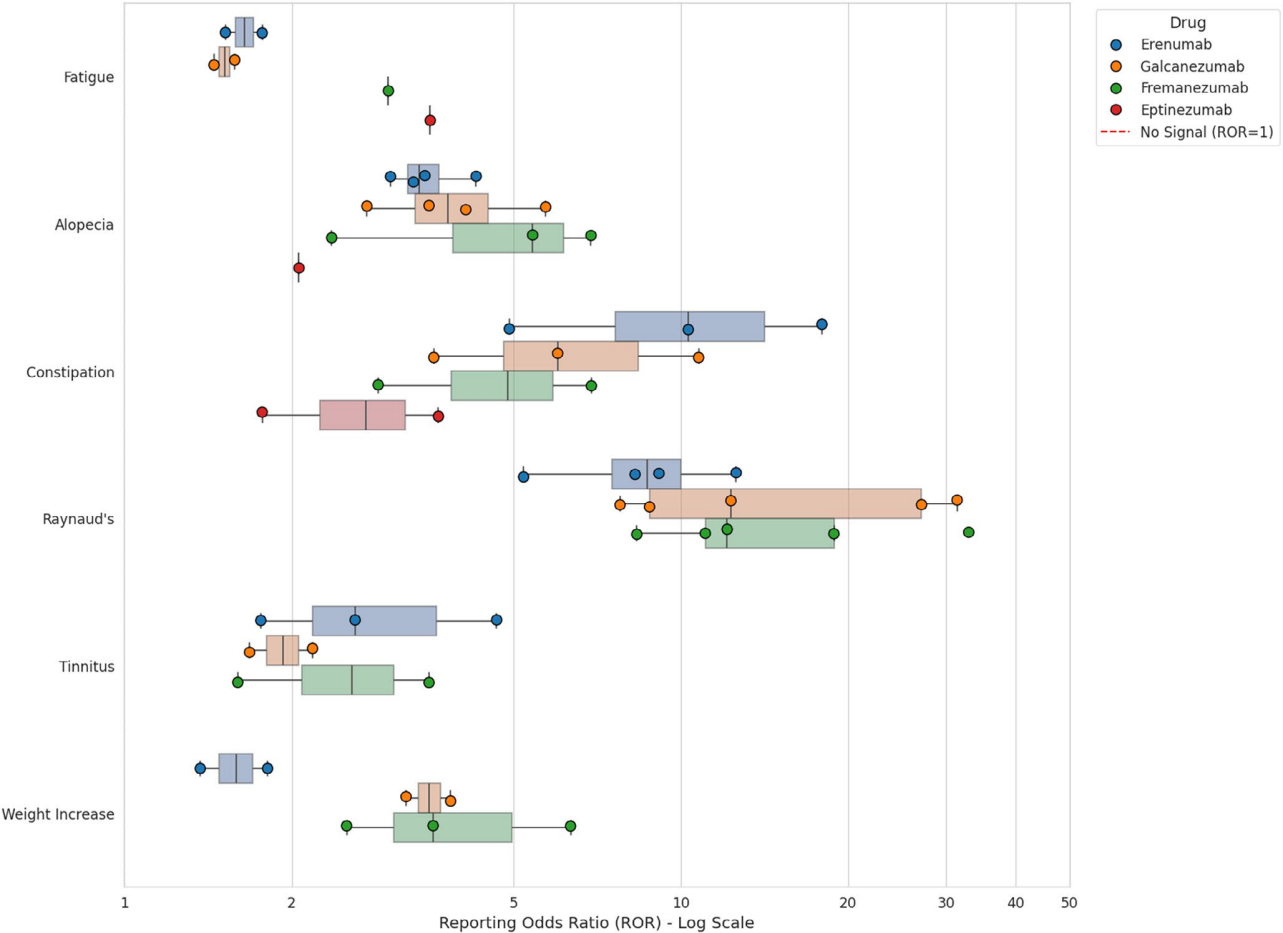
Consistency and magnitude of safety signals (SDRs) for anti‐CGRP monoclonal antibodies. This range plot illustrates the distribution of RORs for key adverse events (fatigue, alopecia, constipation, Raynaud's phenomenon, tinnitus, and weight increase) identified across the included pharmacovigilance studies. Each colored dot represents a specific point estimate (ROR) extracted from an individual study, allowing for a visual assessment of reproducibility. The shaded areas (boxes) visualize the range and distribution of the data, highlighting the degree of heterogeneity among different analyses. Note that a logarithmic scale is used on the x‐axis. The red dashed vertical line at x = 1 represents the threshold for a signal of disproportionate reporting (values to the right indicate a positive association). The plot demonstrates high consistency for alopecia signals across different studies and agents, while revealing significant variability (heterogeneity) in the magnitude of signals for Raynaud's phenomenon and constipation. MAbs, monoclonal antibodies; ROR, reporting odds ratio; SDR, signal of disproportionate reporting. [Color figure can be viewed at wileyonlinelibrary.com]

The analysis of AEs associated with with the four FDA‐approved monoclonal antibodies (erenumab, galcanezumab, fremanezumab, and eptinezumab) confirmed SDRs for common, expected events. An SDR for fatigue was observed for all four agents (Figure 2), with RORs ranging from 1.45 to 3.54 across studies.^40^, ^42^ Injection site reactions also showed strong SDRs for the three subcutaneously administered drugs (Table S4), particularly swelling with fremanezumab (ROR 222.62) and pain with galcanezumab (ROR = 33.90).^42^ A similar SDR for an infusion‐related reaction (IRR) was identified for eptinezumab (ROR = 3.04).^42^

Several significant post‐marketing SDRs emerged, revealing both class‐wide trends and drug‐specific profiles. As shown in Figure 2, alopecia was consistently observed across all four mAbs, with strong SDRs reported in multiple studies, such as a ROR of 6.9 for fremanezumab and 5.72 for galcanezumab.^24^, ^40^ A particularly strong SDR for constipation was identified (Figure 2), primarily for the CGRP receptor antagonist erenumab, with ROR values reaching up to 17.94.^14^, ^42^ While constipation was also identified as an SDR for the three ligand‐directed mAbs, its magnitude was generally lower.^14^, ^40^, ^42^

The analyses also revealed numerous cardiovascular SDRs. Raynaud's phenomenon emerged as a strong and consistent SDR for the subcutaneously administered mAbs—erenumab, galcanezumab, and fremanezumab—with ROR values showing a wide distribution across studies (ranging from ~5 to >30, Figure 2), but it was not found for eptinezumab.^14^, ^22^, ^34^, ^41^, ^42^, ^46^ Hypertension SDRs were identified for all four drugs (Table S4).^21^, ^35^, ^37^, ^42^ Other cardiovascular SDRs included pulmonary embolism for erenumab,^23^ deep vein thrombosis for fremanezumab,^35^ myocardial infarction for erenumab and galcanezumab,^35^, ^42^ and atrial fibrillation for galcanezumab.^35^ Palpitations emerged as an SDR for all four mAbs.^35^, ^40^, ^42^ Further post‐marketing analyses have revealed other consistent SDRs, such as tinnitus and weight increase, which have been reported for erenumab, galcanezumab, and fremanezumab (Table S4).^39^, ^40^, ^42^

Regarding rare but serious cerebrovascular events, distinct SDRs were identified for specific mAbs. For erenumab, a significant SDR was found for reversible cerebral vasoconstriction syndrome (RCVS) (ROR = 9.43).^15^ A class‐level SDR for cerebral and cervical artery dissection (CeAD) was observed (ROR = 7.06), with a particularly strong SDR for galcanezumab (ROR = 14.0).^44^ For eptinezumab, a rare SDR of increased intracranial pressure was observed.^37^ In addition to these specific events, several analyses identified SDRs for cerebrovascular accident associated with erenumab, fremanezumab, and galcanezumab.^28^, ^40^, ^42^ Finally, a comprehensive analysis of the VigiBase database identified a significant class‐level SDR for cerebrovascular diseases for the entire CGRP antagonist class (ROR = 1.22), although this study did not provide data stratified by individual drug.^38^

Finally, the safety profile in pregnancy has also evolved. An initial analysis suggested a class‐level SDR for spontaneous abortion with anti‐CGRP mAbs.^19^ However, subsequent larger analyses did not confirm this SDR, instead suggesting no increased reporting risk compared to triptans.^26^, ^32^ Nevertheless, a more recent analysis of the EudraVigilance database identified specific SDRs for spontaneous abortion with galcanezumab (ROR = 2.30) and eptinezumab (ROR = 5.72), suggesting the risk may not be a uniform class effect and could vary between individual drugs (Table S4).^42^

#### Post‐marketing safety profile of Gepants

**FIGURE 3:**
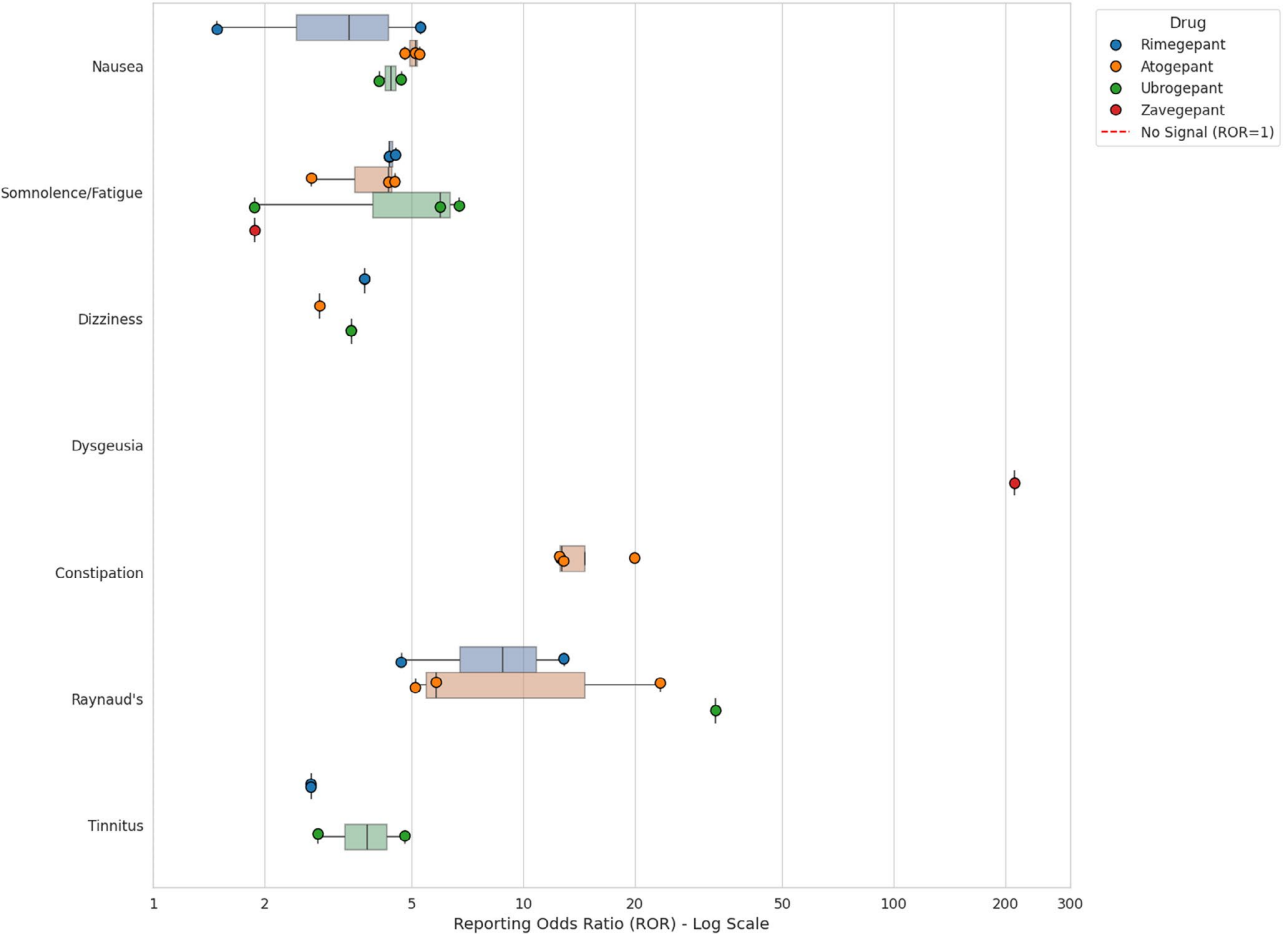
Consistency and magnitude of safety signals (SDRs) for gepants. This range plot illustrates the distribution of RORs for key adverse events (nausea, somnolence/fatigue, dysgeusia, constipation, Raynaud's phenomenon, dizziness, and tinnitus) identified across the included pharmacovigilance studies. Each colored dot represents a specific point estimate (ROR) extracted from an individual study. The shaded areas (boxes) visualize the range and distribution of the data, highlighting the degree of heterogeneity among different analyses. Note that a logarithmic scale is used on the x‐axis to accommodate the exceptionally high magnitude of the signal for dysgeusia associated with zavegepant (linked to its intranasal administration). The red dashed vertical line at x = 1 represents the threshold for a signal of disproportionate reporting (values to the right indicate a positive association). The plot highlights the distinct profile of zavegepant compared to oral gepants (rimegepant, atogepant, ubrogepant), particularly regarding dysgeusia, as well as the variability in vascular signals (Raynaud's phenomenon) across the class. ROR, reporting odds ratio; SDR, signal of disproportionate reporting. [Color figure can be viewed at wileyonlinelibrary.com]

For the gepant class (Figure 3 and Table S5), the analyzes confirmed SDRs for frequent, labeled AEs such as nausea, fatigue/somnolence, and dizziness for rimegepant, atogepant, and ubrogepant, with SDRs for nausea and fatigue also reported for zavegepant.^29^, ^33^, ^40^, ^43^ Further different safety profiles emerged from the post‐marketing analyses. As visually highlighted in Figure 3, a uniquely strong and specific SDR for dysgeusia (altered taste) was identified for the intranasal gepant, zavegepant (ROR_025_ = 212.07).^43^ For atogepant, constipation was the most prominent SDR (Figure 3, ROR_025_ = 19.99), indicating possible problems with tolerability.^30^, ^43^

Similar to mAbs, Raynaud's phenomenon was also found to be a significant SDR in oral gepants, showing considerable heterogeneity across studies as depicted in Figure 3, with a strong disproportionality reported for rimegepant (ROR 12.87), atogepant (ROR 23.45), and ubrogepant (ROR 33.03).^41^ Another comprehensive FAERS analysis further corroborated this association, identifying significant SDRs for both rimegepant (ROR 4.68) and atogepant (ROR 5.11).^46^ In addition, alopecia was identified as a novel AE associated with rimegepant and atogepant, but not ubrogepant (Table S5).^31^

Cardiovascular SDRs appeared to be different for each gepant (Table S5), with SDRs for atrial fibrillation (ROR 10.99) and aneurysm (ROR = 5.31) with rimegepant,^25^, ^33^ cardiac flutter with atogepant (ROR 5.30),^36^ and palpitations with ubrogepant (ROR 3.41),^29^ for which a strong SDR for pallor was also noted (ROR 33.40).^25^ In a crucial difference to the class of mAbs, no significant SDRs for serious cerebrovascular events such as RCVS were identified for any gepant.^15^ However, a large‐scale analysis of the VigiBase database did find a significant SDR for cerebrovascular diseases for the CGRP antagonist class as a whole (which includes gepants) (ROR 1.22), though data were not stratified by individual gepants.^38^ This class‐level finding is supported by a separate analysis that identified an SDR for cerebrovascular accident with rimegepant, atogepant, and ubrogepant.^43^ A wide range of other SDRs were identified in the nervous system, psychiatric, and gastrointestinal tracts (Table S5). For rimegepant, these included feeling abnormal (ROR_025_ = 6.46),^43^ motion sickness,^30^ and a variety of other central nervous system (CNS) and psychiatric effects such as petit mal epilepsy (ROR = 13.74), emotional distress (ROR = 9.42), and panic reaction (ROR = 7.32),^25^ as well as tinnitus (ROR 2.67).^39^ In atogepant, unexpected psychiatric SDRs such as thoughts of self‐harm and brain neoplasms were found,^45^ along with SDRs for brain fog, disturbance in attention, and euphoric mood.^36^ In ubrogepant, notable SDRs included a particularly strong signal for visual hallucinations (ROR 44.55)^25^ and oral paresthesia (ROR 12.71).^29^ Notably, various other gastrointestinal SDRs were reported, such as stomatitis and gastritis for rimegepant, dysphagia for ubrogepant,^25^ and impaired gastric emptying for atogepant.^36^ Finally, regarding safety in special populations, a comprehensive analysis of VigiBase found no disproportionate reporting SDR for pregnancy‐related safety reports for the gepant class compared to triptans (ROR for any pregnancy outcomes since 2018: 0.83, 95% confidence interval [CI] 0.61–1.13).^32^ This broad, class‐level finding offers some reassurance, although it is important to note that analyses of individual drugs have identified specific reporting patterns that require cautious interpretation. For example, SDRs have been observed for reporting terms such as exposure during pregnancy with rimegepant and pregnancy with atogepant, which reflect the circumstances of use rather than specific adverse outcomes.^33^, ^36^, ^45^ Notably, one analysis reported an exceptionally strong statistical SDR for habitual abortion with ubrogepant.^31^ However, this finding should be interpreted with extreme caution for two reasons: it is based on a very low number of cases (*n* = 13), and even the researchers who reported it did not discuss its clinical significance. This highlights the risk of over‐interpreting data from rare events.

## DISCUSSION

This systematic review summarizes the results of 30 pharmacovigilance studies and provides a comprehensive and up‐to‐date overview of the real‐world safety profiles of CGRP mAbs and gepants. The results confirm that, while these therapies are generally well tolerated, they are associated with a nuanced and clinically relevant spectrum of AEs that vary both between and within classes. A formal quality assessment showed that 14 of the 30 included studies were of high quality and seven were of moderate quality, providing a solid basis for this analysis. Despite limitations in a subset of studies, the main strength of this review lies in the synthesis of data from numerous independent analyzes, allowing a robust assessment of signal consistency across different statistical methods. We acknowledge that the databases analyzed (FAERS, EudraVigilance, VigiBase) are not mutually exclusive, as VigiBase aggregates global data. However, the fact that an association between a drug and an event is established by several independent research groups using different cleaning and analytical strategies considerably strengthens the robustness of these SDRs. Indeed, several studies using FAERS and VigiBase independently reported a significant disproportionality for Raynaud's phenomenon with both mAbs and oral gepants.^14^, ^22^, ^34^, ^41^, ^46^ This replication strongly suggests a true mechanism‐based class effect related to the vasoactive role of CGRP.^47^ Similarly, alopecia was shown to be a robust SDR for all four mAbs in several analyzes.^12^, ^24^, ^27^, ^40^ In addition, this SDR was identified as a novel potential AE associated with rimegepant and atogepant, suggesting that it may represent a broader, albeit less pronounced, class‐wide effect.^31^ A similar cross‐class pattern, though with varying strength, is emerging for tinnitus, which has been reported as an SDR for three of the four mAbs and for rimegepant.^39^, ^40^

This review also highlights how the understanding of a potential safety signal can evolve over time and shows the heterogeneity of results. The safety profile of mAbs in pregnancy is a prime example of the evolution of a signal. An initial analysis of VigiBase data revealed a grade‐level SDR for spontaneous abortion,^19^ a finding that was supported by a subsequent analysis of the EudraVigilance database.^23^ However, this initial finding was later refuted by the original research group in a series of increasingly comprehensive analyses. Their work culminated in a 2024 study that, building on their 2023 findings, confirmed there was no SDR for pregnancy‐related safety reports for either the mAb or gepant class when compared to triptans.^26^, ^32^ This demonstrates the importance of continuous pharmacovigilance, as early signals can be refined or refuted as more data are collected post‐marketing. This complexity is also mirrored in the safety data for gepants in pregnancy. While a comprehensive class‐level analysis showed no disproportionate reporting compared to triptans,^32^ indicating that no statistical signal was detected at the class level, analyses of individual drugs present a mixed picture. Notably, supporting the class‐level findings, some studies found no SDRs related to pregnancy for rimegepant and atogepant^31^ or for ubrogepant.^29^ In contrast, other analyses reported SDRs for terms such as “exposure during pregnancy;”^33^, ^36^, ^45^ however, it is important to recognize that these terms indicate the circumstance of drug use rather than a confirmed adverse outcome. The only SDR of a potential AE was an exceptionally strong but statistically fragile signal for the specific MedDRA Preferred Term (PT) “habitual abortion” (clinically referring to recurrent spontaneous miscarriage) with ubrogepant. It is important to contextualize this finding: the signal was based on a very low number of cases (*n* = 13) and was neither clinically interpreted by the study's authors^31^ nor confirmed by mechanistic studies. Because pharmacovigilance data cannot prove causality and this term represents a reporting category rather than a verified clinical diagnosis, this isolated finding highlights that, while the overall data appear reassuring, interpreting SDRs from individual studies – especially those based on rare events – requires significant caution to avoid unwarranted alarm. Heterogeneity is also striking in the context of serious cerebrovascular events. A significant SDR for RCVS was specifically associated with erenumab (ROR 9.43),^15^ while a strong SDR for CeAD was found for galcanezumab (ROR 14.0).^44^ However, this latter finding must be interpreted with caution. Since the analysis compared galcanezumab users to the general population and migraine itself is a risk factor for CeAD, the elevated ROR is likely influenced by confounding by indication (underlying disease bias). In contrast, no significant SDRs for RCVS or CeAD were identified for any of the gepants, representing a critical point of differentiation between the two subclasses that could influence treatment decisions in patients with underlying vascular risks. However, it is important to note that a large‐scale VigiBase analysis did identify a significant, albeit modest, class‐level SDR for cerebrovascular diseases for the entire CGRP antagonist class as a whole (ROR 1.22), though this study did not stratify data by individual drug or subclass.^38^

Aside from cross‐ class trends, this synthesis highlights distinct differences in safety profiles within each subclass, suggesting drug‐specific effects. Among the mAbs, constipation is a particularly striking SDR for the CGRP receptor antagonist erenumab (ROR 10.32), a finding that has been consistently reported in numerous studies,^14^, ^28^, ^40^ and which reinforces a well‐established safety issue that had already prompted a label update to warn of serious complications.^48^ While this is also a SDR for the ligand‐targeted mAbs, it is on a much smaller scale. The gepant class exhibits even greater diversity. A uniquely strong and specific SDR for dysgeusia (altered taste) was identified for the intranasal gepant, zavegepant (ROR_025_ = 212.07).^43^ Conversely, severe constipation proved to be the most significant SDR for atogepant (ROR_025_ = 19.99), accompanied by a strong signal for discontinuation of therapy (ROR 16.58), indicating potential problems with tolerability.^40^, ^43^ These different profiles suggest that mechanisms beyond simple CGRP receptor antagonism, such as off‐target effects, drug formulation, or pharmacokinetic properties, are likely to play an important role. This is also evident in the distinct cardiovascular profiles, with SDRs for atrial fibrillation and aneurysm noted for rimegepant,^25^, ^33^ cardiac flutter for atogepant,^36^ and strong SDRs for palpitations and pallor for ubrogepant.^25^, ^29^

From a clinical perspective, these findings have a direct impact on patient treatment. For example, a physician might prefer a ligand‐targeted mAb (fremanezumab) over a receptor‐targeted mAb (erenumab) in a patient with a history of severe constipation. The rare but serious signals of hypertension and cerebrovascular events with certain mAbs emphasize the need for vigilant monitoring of blood pressure, especially in patients with pre‐existing cardiovascular risk factors.^49^ Specifically, the SDR for hypertension with erenumab, the SDRs for myocardial infarction and atrial fibrillation with galcanezumab, and the risk of RCVS with erenumab or CeAD with galcanezumab require careful consideration when selecting a therapy for patients with relevant comorbidities. While these differences among mAbs are significant, the gepant class exhibits an even greater diversity in its safety profile, offering further opportunities for personalized medicine. For example, a patient experiencing CNS side effects such as fatigue with an oral gepant like rimegepant might be a suitable candidate for zavegepant, which has a distinct profile dominated by localized dysgeusia. Similarly, distinct cardiovascular SDRs, such as atrial fibrillation with rimegepant or pallor with ubrogepant, may influence treatment choice in patients with a history of arrhythmias or specific vascular concerns. Beyond managing these specific medical risks, proactive counseling about the potential for non‐serious but consequential side effects such as alopecia is critical, as they can significantly impact mental health and treatment adherence.^50^

While this systematic review provides valuable insights by synthesizing evidence from multiple sources, its conclusions are inevitably shaped by the inherent limitations of pharmacovigilance data and the SRSs analyzed.^51^ Consequently, the significant heterogeneity observed across studies warrants careful interpretation as it likely stems from several distinct factors rather than biological differences alone. First, database biases play a major role; for instance, FAERS is heavily influenced by U.S. reporting culture and consumer reports, whereas EudraVigilance relies more on European healthcare professionals.^52^ Second, the “Weber effect”—a spike in reporting immediately following regulatory approval—and the differing launch timings of mAbs versus gepants complicate direct comparisons.^53^ Third, notoriety bias likely inflated reporting for specific events following media attention or label updates, such as the addition of warnings for constipation with erenumab.^20^ Fourth, methodological variability is a key driver of heterogeneity, including differences in statistical algorithms (e.g., varying CIs for ROR), detection thresholds, and, critically, the choice of comparator groups (e.g., “all other drugs” versus active comparators like triptans).^54^ This variation in denominators profoundly influences the magnitude of the reported disproportionality SDRs. Finally, differential patient exposure and prescribing trends mean that second‐line users (often prescribed gepants) may have different baseline risk profiles than those starting mAbs.^55^ Fundamentally, it must be emphasized that the SDRs identified in this review represent statistical associations serving as hypothesis‐generating findings, rather than definitive evidence of causal links. The most important limitation is that SRSs cannot be used to determine the true incidence of AEs because they lack a reliable denominator for exposed patients. This fundamental weakness precludes the establishment of definitive causality and the calculation of relative risk.^54^ To remedy this, future prospective, observational cohort studies and analyzes of large claims databases (which contain real‐world data from health insurers linking prescriptions to diagnoses) are essential to quantify the absolute risk and incidence of major AEs such as alopecia, constipation, and Raynaud's phenomenon. A structural limitation regarding data independence must also be acknowledged. There is a hierarchical relationship and significant overlap among the included databases, as VigiBase aggregates global reports, including those from FAERS and EudraVigilance. Furthermore, as shown in Table 1, the observation periods of many studies overlap. Therefore, the consistency of findings described in this review should not be interpreted as validation from independent patient populations, but rather as stable signals across different database views. This confirms that the SDRs are robust enough to persist regardless of the specific database slice or statistical methodology applied.

Another limitation concerns the methodological quality assessment. The included studies were assessed using a checklist adapted from STROBE and RECORD‐PE guidelines. It is important to note that STROBE is primarily a reporting guideline, not a risk‐of‐bias assessment tool. Consequently, a study rated as “High Quality” based on its reporting completeness may still be subject to inherent pharmacovigilance biases (such as the Weber effect or notoriety bias). Additionally, the restriction of this review to published literature introduces a potential publication bias, as pharmacovigilance analyses yielding negative results (i.e., finding “no signal”) are less likely to be published than those reporting positive associations. Furthermore, as this specific tool has not been formally validated, the quality classification should be interpreted with this limitation in mind and restricts comparability with reviews using established risk‐of‐bias tools (e.g., ROBIS).^56^ Regarding future research, the heterogeneity of safety profiles underscores the need for head‐to‐head comparative studies to assess the safety of CGRP inhibitors, which are currently lacking.^57^, ^58^ Studies directly comparing agents with different safety SDRs (e.g. erenumab vs. atogepant in constipation) or comparing the vascular safety of all available agents would be invaluable. Additionally, the biological mechanisms linking CGRP blockade to events such as RCVS and alopecia are not yet fully understood. Further basic and translational research is needed to elucidate these mechanisms, which could pave the way for safer molecules in the future. Finally, targeted long‐term safety monitoring is crucial, especially in populations normally excluded from pivotal trials. This applies not only patients with highly resistant forms of migraine^59^ but also to pediatric patients, pregnant and lactating women, and individuals with significant pre‐existing comorbidities.

## CONCLUSIONS

This systematic review of pharmacovigilance data goes beyond a simple confirmation of the generally manageable safety profile of CGRP inhibitors and reveals a complex and actionable landscape of potential AEs. Crucially, our synthesis draws a critical line of distinction: while rare but serious cerebrovascular events such as reversible cerebral vasoconstriction syndrome and cervical artery dissection have been associated with certain mAbs, this specific association has not been established for the gepant class. This divergence is tempered by a class‐wide SDR for cerebrovascular diseases as a whole and is balanced by common mechanism‐based adverse effects—most notably Raynaud's phenomenon and alopecia—that transcend both drug classes and suggest common mechanistic effects. Furthermore, the considerable heterogeneity observed within each subclass challenges the notion of CGRP inhibitors as a monolithic category. The different safety profiles, ranging from the pronounced constipation SDR of erenumab to the unique dysgeusia of zavegepant, provide a clear rationale for a more personalized risk assessment. This nuanced understanding allows physicians to tailor their treatment decisions to the individual patient profile and ultimately improve patient safety through proactive monitoring and counseling.

## AUTHOR CONTRIBUTIONS

**Martina Giacon:** Conceptualization; data curation; formal analysis; investigation; methodology; visualization; validation; writing – original draft; writing – review and editing. **Salvatore Terrazzino:** Conceptualization; data curation; formal analysis; investigation; methodology; project administration; supervision; visualization; writing – original draft; writing – review and editing.

## FUNDING INFORMATION

No funding was received for this work.

## CONFLICT OF INTEREST STATEMENT

**Martina Giacon** and **Salvatore Terrazzino** have no conflicts of interest to disclose.

## Supporting information


Data S1:
