# Supplementary material for: Post‐marketing safety of CGRP monoclonal antibodies and gepants: A systematic review of spontaneous reporting system data
Source: Headache. 2026 Mar 15;66(5):1128–47. doi: 10.1111/head.70081 (PMC13142213; doi:10.1111/head.70081)
Supplement: Supplementary file 1 — Data S1: [file HEAD-66-1128-s001.docx]

**Supplementary Fig. 1**

Characteristics of Included Pharmacovigilance Studies. Number of studies by (A) publication year; (B) database analyzed; (C) country of corresponding author.


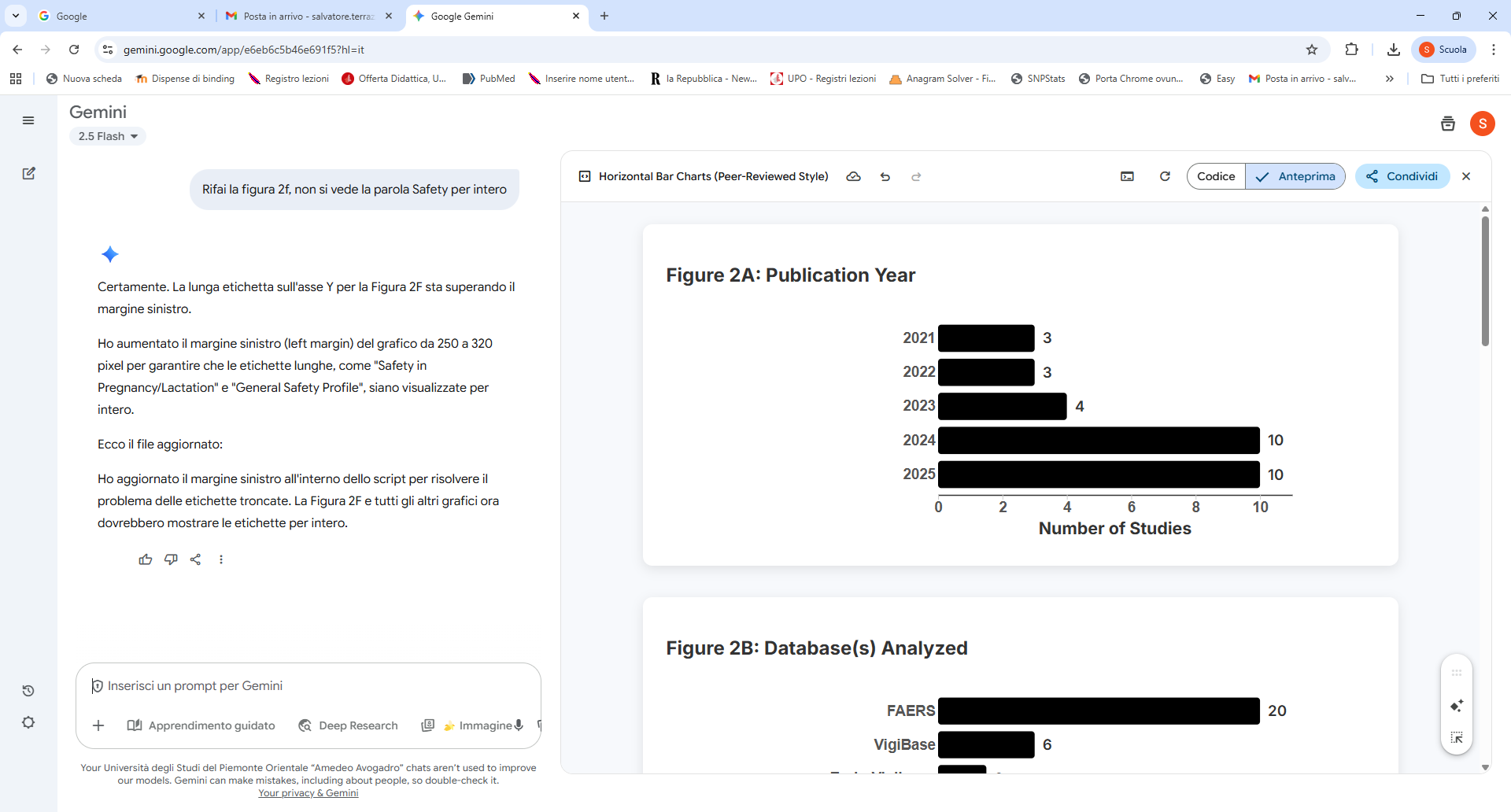

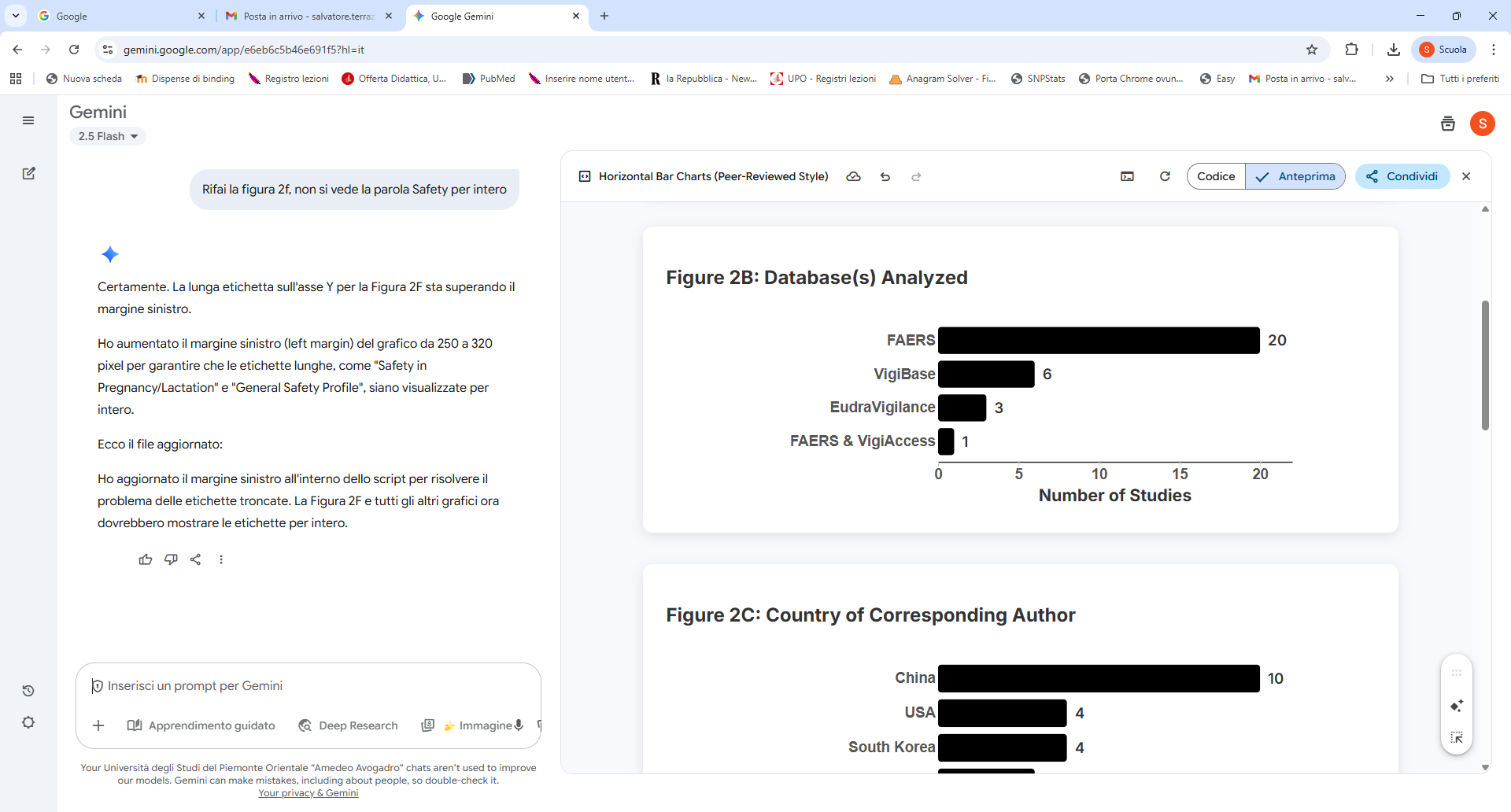

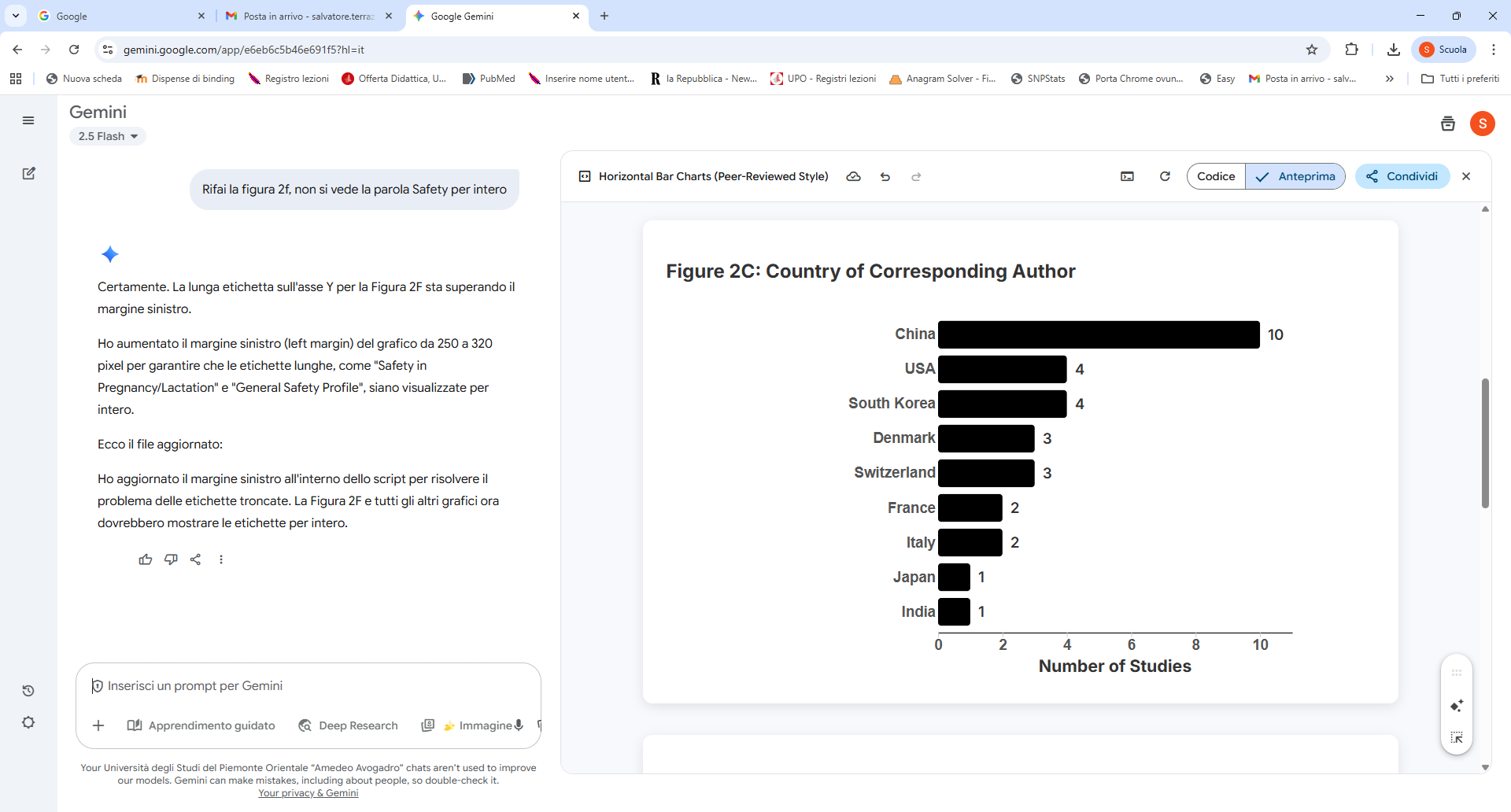


A

B

C

**Supplementary Fig. 2**

Characteristics of Included Pharmacovigilance Studies. Number of studies by (A) primary drug class focus; (B) specific CGRP inhibitors; (C) primary AE or safety focus.


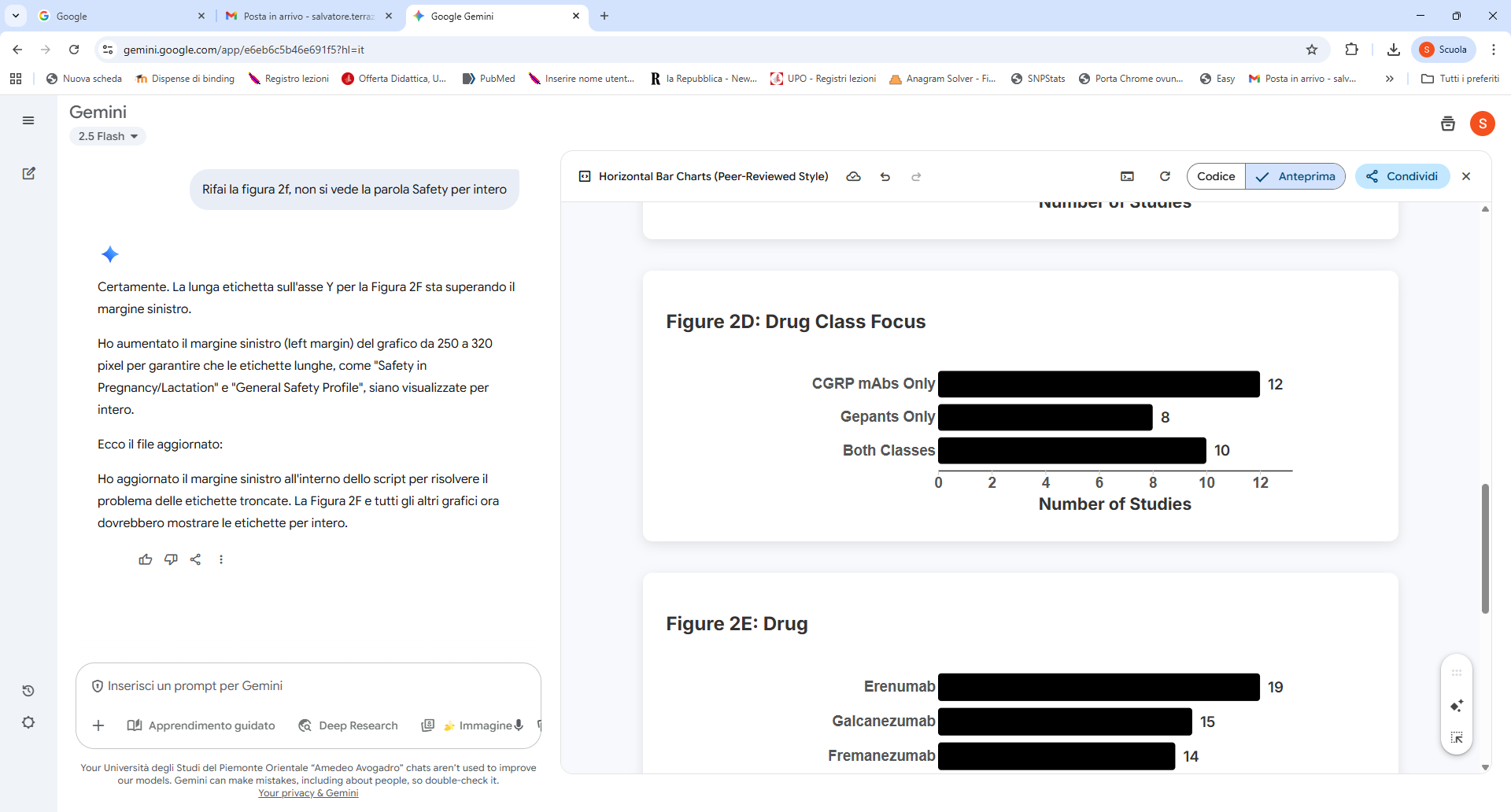

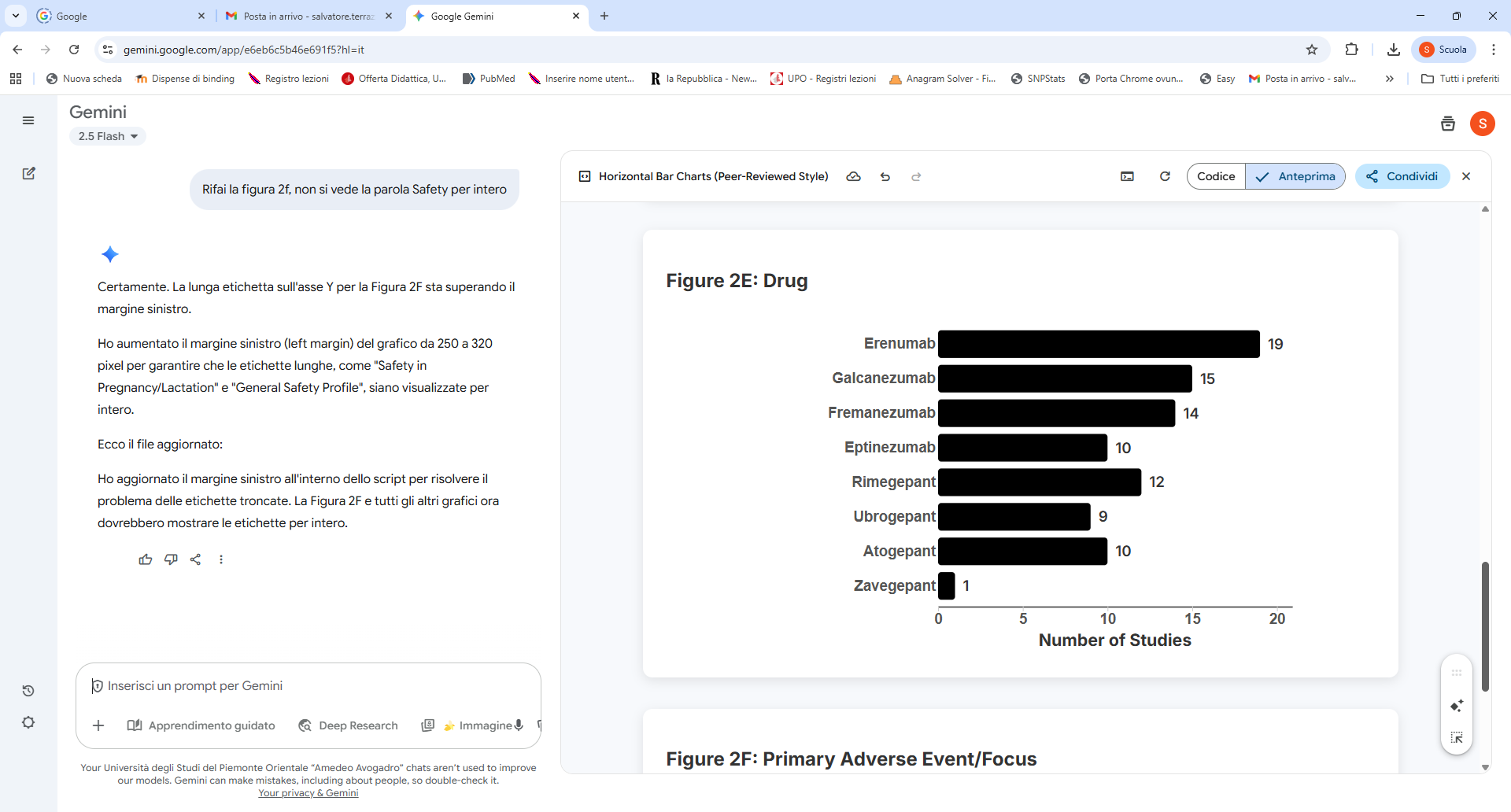

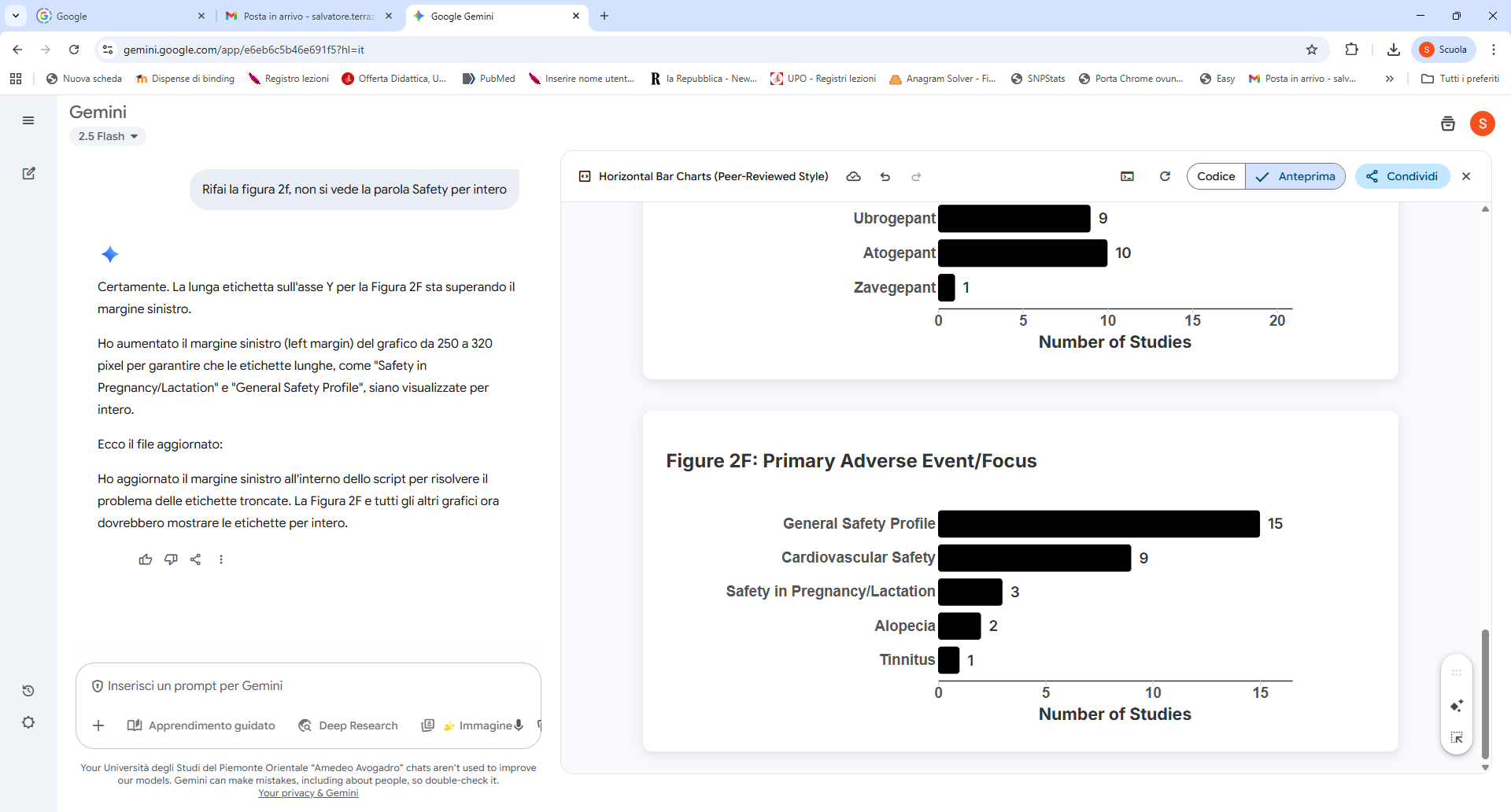


A

B

C


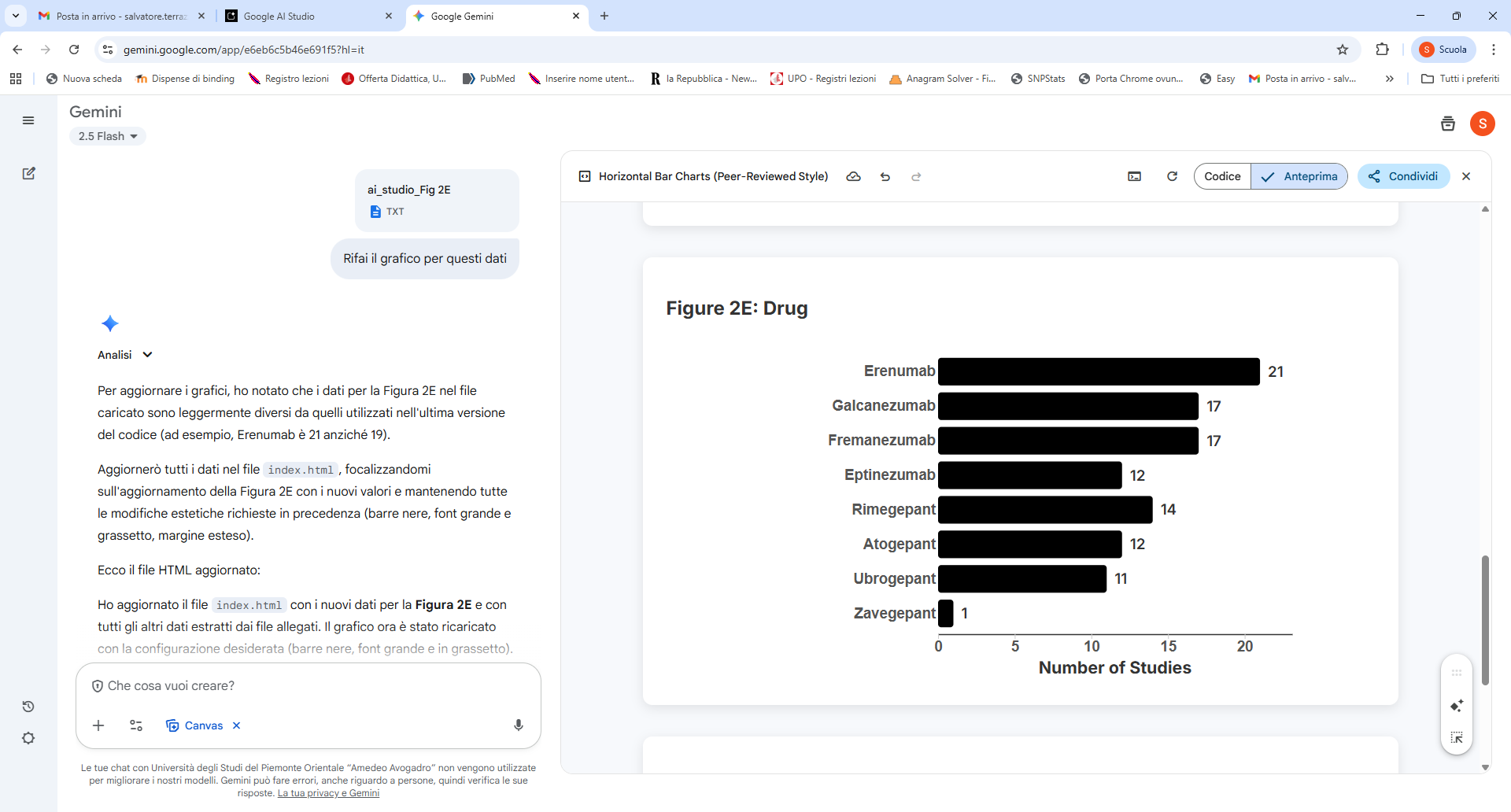


**Supplementary Table 1.** Detailed Search Strategy.

| **Database** | **Interface/URL** | **Search Fields** | **Exact Search String** |
| --- | --- | --- | --- |
| **PubMed** | NCBI | Title/Abstract | (FAERS OR VigiBase OR EudraVigilance OR Pharmacovigilance OR adverse event reporting OR spontaneous reporting) AND (CGRP inhibitor OR CGRP inhibitors OR CGRP antagonist OR CGRP antagonists OR anti-CGRP monoclonal antibody OR anti-CGRP monoclonal antibodies OR gepant OR gepants OR Erenumab OR Aimovig OR Galcanezumab OR Emgality OR Fremanezumab OR Ajovy OR Eptinezumab OR Vyepti OR Rimegepant OR Nurtec OR Vydura OR Ubrogepant OR Ubrelvy OR Atogepant OR Qulipta OR Zavegepant OR Zavzpret) |
| **Web of Science** | Clarivate | Topic (Title, Abstract, Keywords) | Same as above |
| **Cochrane Library** | Wiley | Title, Abstract, Keywords | Same as above |
| **OpenGrey** | DANS Mirror (https://lifesciences.datastations.nl/) | All fields | Same as above |

**Supplementary Table 2.** Methodological Quality Assessment Checklist for Pharmacovigilance Studies.

| **Domain** | **Item No.** | **Criterion for Quality Assessment** |
| --- | --- | --- |
| A. Study Design and Objectives | 1 | Is the study objective clearly defined with a specific research question? |
| B. Data Management | 2 | Is the time period of data extraction from the FAERS database clearly specified (e.g., Q1 2014 - Q4 2024)? |
|  | 3 | Is the search strategy for drugs and adverse events transparent and reproducible? |
|  | 4 | Is the MedDRA (Medical Dictionary for Regulatory Activities) level used to define events specified (e.g., Preferred Term, High-Level Term)? |
|  | 5 | Is a method for managing and removing duplicate case reports described? (Major Criterion) |
| C. Statistical Analysis | 6 | Are standard and appropriate statistical methods used for disproportionality analysis (e.g., ROR, PRR, IC)? (Major Criterion) |
|  | 7 | Are confidence intervals (e.g., 95% CI) and clear thresholds for signal significance reported (e.g., N > 3 cases)? |
|  | 8 | Were sensitivity or stratification analyses conducted to explore the robustness of the results (e.g., by sex, age, reporter type)? |
| D. Bias Management | 9 | Do the authors explicitly acknowledge and discuss the inherent limitations of the FAERS database (e.g., reporting bias, lack of denominator)? (Major Criterion) |
|  | 10 | Is the potential impact of relevant confounding factors discussed (e.g., indication bias, polypharmacy)? |
| E. Interpretation | 11 | Are the conclusions appropriately cautious, framed as "signals" or "associations," and avoiding direct causal language? (Major Criterion) |
|  | 12 | Are the results contextualized with other existing scientific evidence (e.g., clinical trials, biological mechanisms)? |
|  |  | **TOTAL SCORE (/24):** |

**Scoring Instructions:** For each item, assign a score based on the degree to which the criterion is met.

- **Yes (2 points):** The criterion is fully and clearly met.
- **Partially (1 point):** The criterion is mentioned or addressed, but with insufficient detail or clarity.
- **No (0 points):** The criterion is not met or not mentioned.

**Not Applicable (NA):** The criterion does not apply to the specific study design; it is excluded from the maximum possible score calculation.

**Final Quality Assessment:**

Based on the total score and the fulfillment of the four **Major Criteria** (Items 5, 6, 9, 11), the study is assigned to one of the following categories:

- **High Quality:** Score ≥ 85% **AND** all 4 Major Criteria are scored as "Yes" (2 points).
- **Moderate Quality:** Score between 60% and 84% **AND** no Major Criterion is scored as "No" (0 points).
- **Low Quality:** Score < 60% **OR** if any single Major Criterion is scored as "No" (0 points).
- **Critically Low:** Score < 40% **AND** two or more Major Criteria are scored as "No" (0 points).

**Supplementary Table 3.** Methodological Quality Assessment of Included Studies.

| **Study (Author & Year)** | **Item 1** | **Item 2** | **Item 3** | **Item 4** | **Item 5** | **Item 6** | **Item 7** | **Item 8** | **Item 9** | **Item 10** | **Item 11** | **Item 12** | **Total score** | **Final Quality**  **Rating** |
| --- | --- | --- | --- | --- | --- | --- | --- | --- | --- | --- | --- | --- | --- | --- |
| Noseda et al.^19^ | 2 | 2 | 2 | 2 | 1 | 2 | 2 | 2 | 2 | 2 | 2 | 2 | 23/24 | Moderate Quality |
| Saely et al.^20^ | 2 | 2 | 2 | 2 | 2 | NA | NA | 2 | 2 | 2 | 2 | 2 | 20/20* | High Quality |
| Sessa and Andersen^21^ | 2 | 2 | 2 | 2 | 2 | 2 | 2 | 2 | 2 | 2 | 2 | 2 | 24/24 | High Quality |
| Gérard et al.^22^ | 2 | 2 | 2 | 2 | 0 | 2 | 2 | 2 | 2 | 2 | 2 | 2 | 22/24 | Low Quality |
| Liang and Sessa^23^ | 2 | 2 | 2 | 2 | 0 | 2 | 2 | 0 | 2 | 2 | 2 | 2 | 20/24 | Low Quality |
| Woods^24^ | 2 | 2 | 2 | 2 | 0 | 2 | 2 | 2 | 2 | 2 | 2 | 2 | 22/24 | Low Quality |
| Battini et al.^25^ | 2 | 2 | 2 | 2 | 2 | 2 | 2 | 0 | 2 | 2 | 2 | 2 | 22/24 | High Quality |
| Noseda et al.^26^ | 2 | 2 | 2 | 2 | 1 | 2 | 2 | 2 | 2 | 2 | 2 | 2 | 23/24 | Moderate Quality |
| Ruiz et al.^27^ | 2 | 2 | 1 | 0 | 0 | NA | NA | 0 | 2 | 2 | 2 | 2 | 13/20* | Low Quality |
| Silberstein et al.^28^ | 2 | 2 | 2 | 2 | 2 | NA | NA | 0 | 2 | 1 | 2 | 2 | 17/20* | Moderate Quality |
| Cao et al.^29^ | 2 | 2 | 2 | 2 | 2 | 2 | 2 | 0 | 2 | 0 | 2 | 1 | 19/24 | Moderate Quality |
| Favrelière et al.^15^ | 2 | 2 | 2 | 2 | 0 | 2 | 2 | 2 | 2 | 2 | 2 | 2 | 22/24 | Low Quality |
| Hu et al.^30^ | 2 | 2 | 2 | 2 | 2 | 2 | 2 | 2 | 2 | 2 | 2 | 2 | 24/24 | High Quality |
| Liang et al.^31^ | 2 | 2 | 2 | 2 | 0 | 2 | 2 | 0 | 2 | 1 | 2 | 2 | 19/24 | Low Quality |
| Noseda et al.^32^ | 2 | 2 | 2 | 2 | 1 | 2 | 2 | 2 | 2 | 2 | 2 | 2 | 23/24 | Moderate Quality |
| Pan and Lin^33^ | 2 | 2 | 2 | 2 | 2 | 2 | 2 | 0 | 2 | 1 | 2 | 1 | 20/24 | Moderate Quality |
| Singh et al.^34^ | 2 | 2 | 2 | 2 | 0 | 2 | 2 | 2 | 2 | 2 | 2 | 2 | 22/24 | Low Quality |
| Sorbara et al.^35^ | 2 | 2 | 2 | 2 | 2 | 2 | 2 | 0 | 2 | 2 | 2 | 2 | 22/24 | High Quality |
| Sun et al.^14^ | 2 | 2 | 2 | 2 | 2 | 2 | 2 | 1 | 2 | 2 | 2 | 2 | 23/24 | High Quality |
| Zhang et al.^36^ | 2 | 2 | 2 | 2 | 2 | 2 | 2 | 0 | 2 | 2 | 2 | 2 | 22/24 | High Quality |
| Chen et al.^37^ | 2 | 2 | 2 | 2 | 2 | 2 | 2 | 2 | 2 | 2 | 2 | 2 | 24/24 | High Quality |
| Cho et al.^38^ | 2 | 2 | 2 | 2 | 0 | 2 | 2 | 2 | 2 | 1 | 2 | 2 | 21/24 | Low Quality |
| Kim et al.^39^ | 2 | 2 | 2 | 2 | 0 | 2 | 2 | 2 | 2 | 2 | 2 | 2 | 22/24 | Low Quality |
| Lee et al.^40^ | 2 | 2 | 2 | 2 | 2 | 2 | 2 | 2 | 2 | 2 | 2 | 2 | 24/24 | High Quality |
| Lee et al.^41^ | 2 | 2 | 2 | 2 | 2 | 2 | 2 | 2 | 2 | 2 | 2 | 2 | 24/24 | High Quality |
| Nikitina et al.^42^ | 2 | 2 | 2 | 2 | 1 | 2 | 2 | 0 | 2 | 2 | 2 | 2 | 21/24 | Moderate Quality |
| Song et al.^43^ | 2 | 2 | 2 | 2 | 2 | 2 | 2 | 2 | 2 | 2 | 2 | 2 | 24/24 | High Quality |
| Tokuyasu et al.^44^ | 2 | 2 | 2 | 2 | 2 | 2 | 2 | 1 | 2 | 2 | 2 | 2 | 23/24 | High Quality |
| Wen et al.^45^ | 2 | 2 | 2 | 2 | 2 | 2 | 2 | 0 | 2 | 1 | 2 | 2 | 21/24 | High Quality |
| Zheng et al.^46^ | 2 | 2 | 2 | 2 | 2 | 2 | 2 | 2 | 2 | 2 | 2 | 2 | 24/24 | High Quality |

**Note: The maximum possible score is 20, as items 6 and 7 related to disproportionality analysis are not applicable (NA) to this study's design.*

**Supplementary Table 4.** Stratified Summary of Adverse Events for Anti-CGRP Monoclonal Antibodies.

| **Category** | **Adverse Event** | **Erenumab** | **Galcanezumab** | **Fremanezumab** | **Eptinezumab** |
| --- | --- | --- | --- | --- | --- |
| **Common, Labeled AEs** | **Injection Site Reactions** | RR = 2.94 per 1000 for injection-site pain (Silberstein et al.^28^); 14.1% of AEs (Sun et al.^14^); ROR = 10.48 (95% CI 6.69-16.42) for injection site pain (Nikitina et al.^42^) | RR = 4.90 per 1000 for injection-site pain (Silberstein et al.^28^); ROR = 33.90 (95% CI 21.64-53.11) for injection site pain (Nikitina et al.^42^) | RR = 0.81 per 1000 for injection-site pain (Silberstein et al.^28^): 13.1% of AEs (Sun et al.^14^); ROR = 222.62 (95% CI 55.25-896.98) for injection site swelling (Nikitina et al.^42^) | ROR = 3.04 (95% CI 1.30-7.13) for infusion-related reaction (Nikitina et al.^42^) |
|  | **Fatigue** | SDR found (Liang and Sessa^23^); RR = 2.33 per 1000 (Silberstein et al.^28^); ROR = 1.52 (95% CI 1.42-1.63) (Lee et al.^40^); ROR = 1.77 (95% CI 1.53-2.03) (Nikitina et al.^42^) | ROR = 1.45 (95% CI 1.29-1.64) (Lee et al.^40^); ROR = 1.58 (95% CI 1.29-1.93) (Nikitina et al.^42^) | ROR = 2.98 (95% CI 2.56-3.47) (Lee et al.^40^) | ROR = 3.54 (95% CI 2.56-4.90) (Nikitina et al.^42^) |
| **AESIs / Post-Marketing Signals** | **Alopecia** | SDR found (Sessa, and Andersen^21^); SDR (Liang and Sessa^23^); PRR = 4.29 (95% CI 4.05-4.54) (Woods^24^); ROR = 3.31 (95% CI 3.12-3.51) (Sun et al.^14^); ROR = 3.47 (95% CI 3.23-3.73) (Lee et al.^40^); ROR = 3.01 (95% CI 2.51-3.60) (Nikitina et al.^42^) | PRR = 4.11 (95% CI 3.78-4.48) (Woods^24^); ROR = 3.53 (95% CI 3.25-3.83) (Sun et al.^14^); ROR = 5.72 (95% CI 5.09-6.43) (Lee et al.^40^); ROR = 2.73 (95% CI 2.16-3.45) (Nikitina et al.^42^) | PRR = 5.42 (95% CI 4.66-6.29) (Woods^24^); ROR = 2.73 (95% CI 2.36-3.16) (Sun et al.^14^); ROR = 6.9 (95% CI 5.72-8.33) (Lee et al.^40^) | PRR = 2.06 (95% CI 1.25-3.40) (Woods^24^); No SDR found (Sun et al.^14^) |
|  | **Constipation** | RR = 4.90 per 1000 (Silberstein et al.^28^); ROR = 10.32 (95% CI 9.94-10.72) (Sun et al.^14^); ROR = 4.92 (95% CI 4.46-5.42) (Lee et al.^40^); ROR = 17.94 (95% CI 13.85-23.24) (Nikitina et al.^42^) | ROR = 3.60 (95% CI 3.30-3.94) (Sun et al.^14^); ROR = 6.01 (95% CI 5.35-6.75) (Lee et al.^40^); ROR = 10.77 (95% CI 8.06-14.40) (Nikitina et al.^42^) | ROR = 2.86 (95% CI 2.45-3.35) (Sun et al.^14^); ROR = 6.91 (95% CI 5.71-8.37) (Lee et al.^40^) | ROR = 1.77 (95% CI 1.24-2.52) (Sun et al.^14^); ROR = 3.67 (95% CI 1.93-6.96) (Nikitina et al.^42^) |
|  | **Raynaud's Phenomenon** | IC = 3.2 (95% CI 2.8-3.5) (Gérard et al.^22^); ROR = 9.14 (95% CI 6.8-12.28) (Singh et al.^34^); no SDR found (Sorbara et al.^35^); ROR = 8.28 (95% CI 6.24-10.97) (Sun et al.^14^); ROR = 12.58 (95% CI 9.12-17.36) (Lee et al.^41^); ROR = 5.22 (95% CI 4.00-6.81) (Zheng et al.^46^) | IC = 3.2 (95% CI 2.6-3.7) (Gérard et al.^22^); ROR = 8.79 (95% CI 6.05-12.76) (Singh et al.^34^); no SDR found (Sorbara et al.^35^); ROR = 12.31 (95% CI 8.81-17.21) (Sun et al.^14^); ROR = 31.38 (95% CI 21.43-45.94) (Lee et al.^41^); ROR = 27.09 (8.18-89.76) (Nikitina et al.^42^); ROR = 7.78 (95% CI 5.74-10.55) (Zheng et al.^46^) | IC = 3.2 (95% CI 2.3-3.8) (Gérard et al.^22^); ROR = 11.07 (95% CI 5.75-21.32) (Singh et al.^34^); no SDR found (Sorbara et al.^35^); ROR = 12.12 (95% CI 7.16-20.51) (Sun et al.^14^); ROR = 32.93 (95% CI 19.06-56.91) (Lee et al.^41^); ROR = 18.85 (5.68-62.63) (Nikitina et al.^42^); ROR = 8.34 (95% CI 5.02-13.85) (Zheng et al.^46^) | No reports found (Gérard et al.^22^); no SDR found (Sun et al.^14^), no SDR found (Lee et al.^41^); no SDR found (Zheng et al.^46^) |
|  | **Tinnitus** | SDR found (Sessa and Andersen^21^; Liang et al.^23^); ROR = 2.60 (95% CI 2.25-3.00) (Kim et al.^39^); ROR = 4.67 (95% CI 3.94-5.54) (Lee et al.^40^); ROR = 1.76 (95% CI 1.28-2.41) (Nikitina et al.^42^) | ROR = 2.18 (95% CI 1.75-2.73) (Kim et al.^39^); ROR = 1.68 (95% CI 1.08-2.61) (Nikitina et al.^42^) | ROR = 3.53 (95% CI 2.54-4.90) (Kim et al.^39^); ROR = 1.60 (95% CI 1.06-2.41) (Nikitina et al.^42^) | No SDR found (Kim et al.^39^) |
|  | **Weight Increase** | SDR found (Sessa and Andersen^21^; Liang et al.^23^); ROR = 1.37 (95% CI 1.24-1.51) (Sun et al.^14^); ROR = 1.81 (95% CI 1.44-2.29) (Nikitina et al.^42^) | ROR = 3.86 (95% CI 3.54-4.20) (Sun et al.^14^); ROR = 3.21 (95% CI 2.47-4.19) (Nikitina et al.^42^) | ROR = 2.51 (95% CI 2.12-2.96) (Sun et al.^14^); ROR = 6.34 (95% CI 5.18-7.76) (Lee et al.^40^); ROR = 3.59 (95% CI 2.80-4.60) (Nikitina et al.^42^) | No SDR found (Sun et al.^14^) |
|  | **Hypertension & CV Signals** | SDR of elevated blood pressure (Saely et al.^20^); SDR found (Sessa and Andersen^21^); ROR = 10.97 (95% CI 1.35-89.21) for deep-vein thrombosis (Liang and Sessa^23^); SDR found for pulmonary embolism (Liang and Sessa^23^); RR = 0.21 per 1000 for hypertension (Silberstein et al.^28^); ROR = 1.45 (95% CI 1.14-1.85) for hypertension (Sorbara et al.^35^); no SDR found for hypertension (Sun et al.^14^); ROR = 2.67 (95% CI 2.32-3.07) for palpitations (Lee et al.^40^); ROR = 3.25 (95% CI 2.38-4.43) for hypertension (Nikitina et al.^42^) | RR = 0.08 per 1000 for hypertension (Silberstein et al.^28^); ROR = 2.36 (95% CI 1.02-5.46) for atrial fibrillation (Sorbara et al.^35^); no SDR found for hypertension (Sorbara et al.^35^); no SDR found for hypertension (Sun et al.^14^); ROR = 3.79 (95% CI 3.1-4.63) for palpitations (Lee et al.^40^); ROR = 2.75 (95% CI 1.84-4.12) for hypertension (Nikitina et al.^42^) | RR = 0.05 per 1000 for hypertension (Silberstein et al.^28^); ROR = 3.86 (95% CI 1.57-9.51) for deep vein thrombosis (Sorbara et al.^35^); ROR = 1.48 (95% CI 1.05-2.08) for palpitations (Sorbara et al.^35^); no SDR found for hypertension (Sorbara et al.^35^; Sun et al.^14^); ROR = 2.84 (95% CI 1.94-4.15) for hypertension (Nikitina et al.^42^) | No SDR found for hypertension (Sun et al.^14^); ROR = 3.69 (95% CI 3.07-4.42) for hypertension (Chen et al.^37^); ROR = 6.93 (95% CI 3.39-14.18) for palpitations (Nikitina et al.^42^) |
|  | **Serious Cerebrovascular Events** | SDR found (Sessa and Andersen^21^); RR = 0.11 per 1000 for cerebrovascular accident (Silberstein et al.^28^); ROR = 9.43 (95% CI 4.5-19.8) for RCVS (Favrelière et al.^15^); ROR = 1.22 (95% CI 1.12-1.33) for the class of CGRP antagonists (Cho et al.^38^); ROR = 1.43 (95% CI 1.18-1.72) for cerebrovascular accident (Lee et al.^40^); ROR = 2.52 (95% CI 1.67-3.83) for cerebrovascular accident (Nikitina et al.^42^); ROR = 3.64 (95% CI 1.96-6.73) for myocardial infarction (Nikitina et al.^42^); ROR = 7.06 (95% CI 3.75-13.3) for cerebral/cervical artery dissection (Tokuyasu et al.^44^) | RR = 0.05 per 1000 for cerebrovascular accident (Silberstein et al.^28^); ROR = 2.21 (95% CI 1.37-3.58) for myocardial infarction (Sorbara et al.^35^); ROR = 1.22 (95% CI 1.12-1.33) for the class of CGRP antagonists (Cho et al.^38^); ROR = 4.73 (95% CI 2.99-7.47) for cerebrovascular accident (Nikitina et al.^42^); ROR = 6.75 (95% CI 3.48-13.09) for myocardial infarction (Nikitina et al.^42^); ROR = 14.0 (95% CI 6.22-31.4) for cerebral/cervical artery dissection (Tokuyasu et al.^44^) | RR = 0.08 per 1000 for cerebrovascular accident (Silberstein et al.^28^); no SDR found for myocardial infarction (Sorbara et al.^35^); ROR = 1.22 (95% CI 1.12-1.33) for the class of CGRP antagonists (Cho et al.^38^); ROR = 2.20 (95% CI 1.33-3.66) for cerebrovascular accident (Nikitina et al.^42^); ROR = 7.06 (95% CI 3.75-13.3) for cerebral/cervical artery dissection (Tokuyasu et al.^44^) | ROR = 3.47 (95% CI 1.12-10.76) for increased intracranial pressure (Chen et al.^37^); RR = 0.82 (95% CI 0.34-1.95) for cardiovascular or cerebrovascular disorders; no SDR found (Chen et al.^37^); ROR = 1.22 (95% CI 1.12-1.33) for the class of CGRP antagonists (Cho et al.^38^); ROR = 7.06 (95% CI 3.75-13.3) for cerebral/cervical artery dissection (Tokuyasu et al.^44^) |
|  | **Cerebrovascular diseases (general)** | SDR found (Sessa and Andersen^21^); RR = 0.11 per 1000 for cerebrovascular accident (Silberstein et al.^28^); ROR = 1.22 (95% CI 1.12-1.33) for the class of CGRP antagonists (Cho et al.^38^); ROR = 2.52 (95% CI 1.67-3.83) (Nikitina et al.^42^); ROR = 7.06 (95% CI 3.75-13.3) for the class of CGRP mAbs (Tokuyasu et al.^44^) | RR = 0.05 per 1000 for cerebrovascular accident (Silberstein et al.^28^); ROR = 1.22 (95% CI 1.12-1.33) for the class of CGRP antagonists (Cho et al.^38^); ROR = 4.73 (95% CI 2.99-7.47) (Nikitina et al.^42^); ROR = 14.0 (95% CI 6.22-31.4) (Tokuyasu et al.^44^) | RR = 0.08 per 1000 for cerebrovascular accident (Silberstein et al.^28^); ROR = 1.22 (95% CI 1.12-1.33) for the class of CGRP antagonists (Cho et al.^38^); ROR = 2.20 (95% CI 1.33-3.66) (Nikitina et al.^42^); ROR = 7.06 (95% CI 3.75-13.3) for the class of CGRP mAbs (Tokuyasu et al.^44^) | ROR = 1.22 (95% CI 1.12-1.33) for the class of CGRP antagonists (Cho et al.^38^); ROR = 7.06 (95% CI 3.75-13.3) for the class of CGRP mAbs (Tokuyasu et al.^44^) |
|  |  |  |  |  |  |
| **Special Populations** | **Safety in Pregnancy** | ROR = 1.86 (95% CI 1.12-3.13) for spontaneous abortion vs triptans (for anti-CGRP mAbs as a class) (Noseda, et al.^19^); ROR = 2.16 (95% CI 1.13-4.12) for spontaneous abortion (Liang and Sessa^23^); ROR = 1.2 (95% CI 0.8-1.9) vs triptans; not significant (for anti-CGRP mAbs as a class) (Noseda, et al.^26^); ROR = 0.38 (95% CI 0.29-0.50) for any pregnancy outcomes vs triptans (for anti-CGRP mAbs as a class) (Noseda et al.^32^) | ROR = 2.30 (95% CI 1.33-3.98) for spontaneous abortion (Nikitina et al.^42^) |  | ROR = 5.72 (95% CI 1.37-23.97) for spontaneous abortion (Nikitina et al.^42^) |

AEs: Adverse Events; AESIs: Adverse Events of Special Interest; CI: Confidence Interval; CV: Cardiovascular; IC: Information Component; mAb: Monoclonal Antibody; PRR: Proportional Reporting Ratio; RCVS: Reversible Cerebral Vasoconstriction Syndrome; ROR: Reporting Odds Ratio; RR: Reporting Rate; SDR: Signal of Disproportionate Reporting. Note: The magnitude of disproportionality metrics (ROR, PRR, IC) presented in this table varies depending on the comparator group (e.g., all other drugs vs. specific migraine treatments) and statistical method used in the primary studies. Values should be interpreted within the context of the individual study design and not directly compared across different analyses.The term 'SDR found' is used when a source article confirms a statistically significant disproportionality signal but does not report the specific risk metric value (e.g., ROR, IC) and/or its 95% Confidence Interval. The term 'No SDR found' indicates that an analysis was conducted, but the results were not statistically significant. The term 'No reports found' is used when a study explicitly states that no adverse event reports were found for that specific drug-event combination.

**Supplementary Table 5.** Stratified Summary of Adverse Events for Gepants.

| **Category** | **Adverse Event** | **Rimegepant** | **Atogepant** | **Ubrogepant** | **Zavegepant** |
| --- | --- | --- | --- | --- | --- |
| **Common, Labeled AEs** | **Nausea** | SDR found (Battini et al.^25^); SDR found for nausea: a higher likelihood was reported in females compared to males: ROR = 1.49 (95% CI 1.07-2.06) (Hu et al.^30^); ROR = 5.28 (95% CI 4.89-5.71) (Pan and Lin^33^); ROR (95% CI lower 5.07) (Liang et al.^31^); SDR found (Song et al.^43^) | ROR (95% CI lower 4.79) (Liang et al.^31^); ROR = 5.11 (95% CI 4.58-5.69) (Zhang et al.^36^); SDR found (Song et al.^43^); ROR = 5.25 (95% CI 4.70-5.86) (Wen et al.^45^) | SDR found (Battini et al.^25^); ROR = 4.68 (95% CI 3.9-5.62) (Cao et al.^29^); ROR (95% CI lower 4.08) (Liang et al.^31^); SDR found (Song et al.^43^) | SDR found (Song et al.^43^) |
|  | **Fatigue / Somnolence** | Somnolence: SDR found (Battini et al.^25^); somnolence: ROR = 4.35 (95% CI 3.69-5.12) (Pan and Lin^33^); somnolence: ROR = 4.52 (95% CI 3.57-5.73) (Lee et al.^40^); somnolence: SDR found (Song et al.^43^) | Somnolence: ROR = 4.33 (95% CI 3.44-5.44) (Zhang et al.^36^); fatigue: SDR found (Song et al.^43^); somnolence: SDR found (Song et al.^43^); fatigue: ROR = 2.68 (95% CI 2.33-3.08) (Wen et al.^45^); somnolence: ROR = 4.50 (95% CI 3.57-5.68) (Wen et al.^45^) | Somnolence: SDR found (Battini et al.^25^); somnolence: ROR = 6.72 (95% CI 5.02-9) (Cao et al.^29^); somnolence: ROR (95% CI lower 5.97) (Liang et al.^31^); fatigue: ROR₀₂₅ = 1.88 (Song et al.^43^); somnolence: SDR found (Song et al.^43^) | Fatigue: SDR found (Song et al.^43^) |
|  | **Dizziness** | SDR found (Battini et al.^25^); SDR found (Pan and Lin^33^); ROR = 3.73 (95% CI 3.15-4.42) (Lee et al.^40^) | ROR = 2.81 (95% CI 2.34-3.38) (Wen et al.^45^) | SDR found (Battini et al.^25^); ROR = 3.43 (95% CI 2.63-4.47) (Cao et al.^29^) | SDR found (Song et al.^43^) |
| **AESIs / Post-Marketing Signals** | **Dysgeusia (Altered Taste)** | No SDR found (Pan and Lin^33^); SDR found (Song et al.^43^) | No SDR found (Wen et al.^45^) |  | ROR₀₂₅ = 212.07 (Song et al.^43^) |
|  | **Constipation** | No SDR found (Pan and Lin^33^); SDR found (Song et al.^43^) | ROR (95% CI lower 12.61) (Liang et al.^31^); ROR = 12.53 (95% CI 11.07-14.19) (Zhang et al.^36^); ROR₀₂₅ = 19.99 (Song et al. ^43^); ROR = 12.86 (95% CI 11.32-14.60) (Wen et al.^45^) | SDR found (Song et al.^43^) |  |
|  | **Raynaud's Phenomenon** | No SDR found (Gérard et al.^22^); ROR = 5.30 (95% CI 2.2-12.76); ROR (95% CI lower 6.51) (Liang et al.^31^); (Pan and Lin^33^); ROR = 5.477 (95% CI 2.05; 14.62) (Singh et al.^34^); ROR = 12.87 (95% CI 5.34-31.01) (Lee et al.^41);^ ROR = 4.68 (95% CI 2.34-9.37) (Zheng et al.^46^) | No reports found (Gérard et al.^22^); no reports found (Singh et al.^34^); ROR (95% CI lower 5.98) (Liang et al.^31^); ROR = 5.82 (95% CI 1.87-18.08) (Zhang et al.^36^); ROR = 23.45 (95% CI 7.54-73.01) (Lee et al.^41^); ROR = 5.82 (95% CI 1.87-18.08) (Wen et al.^45^); ROR = 5.11 (95% CI 1.91-13.62) (Zheng et al.^46^) | No SDR found (Gérard et al.^22^); no reports found (Singh et al.^34^); ROR = 33.03 (95% CI 10.61-102.81) (Lee et al.^41^); ROR = 33.03 (95% CI 10.61-102.81) (Zheng et al.^46^) |  |
|  | **Tinnitus** | No SDR found (Pan and Lin^33^); ROR = 2.67 (95% CI 1.66-4.30) (Kim et al.^39^) | No SDR found (Kim et al.^39^); no SDR found (Wen et al.^45^) | ROR = 4.79 (95% CI 2.28-10.07) (Cao et al.^29^); ROR = 2.79 (95% CI 1.16-6.71) (Kim et al.^39^) |  |
|  | **Alopecia** | No SDR found (Woods^24^); no SDR found (Ruiz et al.^27^); SDR (Liang et al.^31^); no SDR found (Pan and Lin^33^); SDR found (Song et al.^43^) | No reports found (Woods^24^); no SDR found (Ruiz et al.^27^); SDR found (Liang et al.^31^); SDR found (Song et al.^43^); no SDR found (Wen et al.^45^) | No SDR found (Woods^24^); no SDR found (Ruiz et al.^27^); SDR found (Song et al.^43^) |  |
|  | **Hypertension & CV Signals** | Atrial fibrillation: ROR = 10.99 (95% CI 4.12-29.31) (Battini et al.^25^); aneurysm: ROR = 5.31 (95% CI 1.99-14.19) (Pan and Lin^33^); palpitations: SDR found (Song et al.^43^) | Cardiac flutter: ROR (95% CI lower 4.69) (Liang et al. ^31^); cardiac flutter: ROR = 5.3 (95% CI 1.71-16.47) (Zhang et al.^36^); palpitations: SDR found (Song et al.^43^); cardiac flutter: ROR = 5.3 (95% CI 1.71-16.47) (Wen et al.^45^) | Pallor: ROR = 33.40 (95% CI 6.74-165.55) (Battini et al.^25^); palpitations: ROR 3.41 (95% CI 1.98-5.88) (Cao et al.^29^); palpitations: SDR found (Song et al.^43^) |  |
|  | **Serious Cerebrovascular Events** | No SDR found (Favrelière et al.^15^). The study conducted a disproportionality analysis for signals of RCVS but reported no SDR for rimegepant, atogepant, and ubrogepant. Given zavegepant's approval in March 2023, it is highly unlikely that sufficient adverse event reports were available by the study's data cutoff date of May 31, 2023, to permit a meaningful analysis. | | | |
|  |  | Cerebrovascular accident: SDR found (Song et al.^43^) | Cerebrovascular accident: SDR found (Song et al.^43^); no SDR found (Wen et al.^45^) | Cerebrovascular accident: SDR found (Song et al.^43^) |  |
|  | **Cerebrovascular diseases (general)** |  | No SDR found (Wen et al.^45^) |  |  |
|  |  | A class-level SDR for cerebrovascular diseases was identified for the CGRP antagonist class as a whole (which includes gepants and mAbs) (ROR 1.22; 95% CI 1.12-1.33). The study did not provide data stratified by individual drug (Cho et al.^38^). | | | |
|  | **CNS / Psychiatric Effects** | Petit mal epilepsy: ROR = 13.74 (95% CI 4.49-42.02) (Battini et al.^25^); emotional distress: ROR = 9.42 (95% CI 2.43-36.43) (Battini et al.^25^); hypersomnia: ROR = 3.88 (95% CI 1.91-7.89) (Battini et al.^25^); seizure: ROR = 2.11 (95% CI 1.22-3.65) (Battini et al.^25^); panic reaction: ROR = 7.32 (95% CI 1.98-27.07) (Battini et al. ^25^); feeling jittery: ROR = 5.31 (95% CI 2.32-12.12) (Battini et al.^25^); agitation: ROR = 4.58 (95% CI 1.75-12.01) (Battini et al.^25^); anger: ROR = 3.88 (95% CI 1.14-13.24) (Battini et al.^25^); fear: ROR = 3.79 (95% CI 1.47-9.80) (Battini et al.^25^); panic attack: ROR = 2.24 (95% CI 1.17-4.30) (Battini et al.^25^); paresthesia: ROR = 2.00 (95% CI 1.37-2.93) (Battini et al.^25^); feeling drunk: SDR found (Hu et al.^30^); feeling drunk: ROR (95% CI lower 8.31) (Liang et al.^31^); motion sickness: ROR = 12.66 (95% CI 4.73-33.87) (Pan and Lin^33^); feeling drunk: ROR = 9.63 (95% CI 5-18.55) (Pan and Lin^33^); panic attack: ROR = 3.29 (95% CI 2.07-5.22) (Pan and Lin^33^); fear: ROR = 3.90 (95% CI 2.21-6.88) (Pan and Lin^33^); anxiety: SDR found (Song et al.^43^); insomnia: SDR found (Song et al.^43^) | Brain fog: ROR (95% CI lower 15.5) (Liang et al.^31^); brain fog: ROR = 7.2 (95% CI 4.18-12.43) (Zhang et al.^36^); disturbance in attention: ROR = 3.5 (95% CI 2.14-5.73) (Zhang et al.^36^); migraine: ROR = 80.18 (95% CI 73.97-86.91) (Zhang et al.^36^); headache: ROR = 7.14 (95% CI 6.44-7.91) (Zhang et al.^36^); cluster headache: ROR = 49.2 (95% CI 21.87-110.66) (Zhang et al.^36^); head discomfort: ROR = 6.07 (95% CI 3.44-10.7) (Zhang et al.^36^); migraine with aura: ROR = 21.45 (95% CI 8.88-51.79) (Zhang et al.^36^); tension headache: ROR = 7.56 (95% CI 2.83-20.18) (Zhang et al.^36^); ROR = 23.98 (95% CI 7.68-74.89) (Zhang et al.^36^); nightmare: ROR = 3.67 (95% CI 1.91-7.06) (Zhang et al.^36^); abnormal dreams: ROR = 6.4 (95% CI 3.32-12.32) (Zhang et al.^36^); euphoric mood: ROR = 7.02 (95% CI 2.92-16.9) (Zhang et al.^36^); self-injurious ideation: ROR = 10.19 (95% CI 3.27-31.69) (Zhang et al.^36^); anxiety: disproportionality signal found (Song et al.^43^); insomnia: SDR found (Song et al.^43^); seizure: SDR found (Song et al.^43^); post concussion syndrome: ROR = 109.13 (95% CI 34.00-350.20) (Wen et al.^45^); cluster headache: ROR = 52.71 (95% CI 23.39-118.75) (Wen et al.^45^); self-injurious ideation: ROR = 10.69 (95% CI 3.44-33.28) (Wen et al.^45^); tension headache: ROR = 7.77 (95% CI 2.91-20.75) (Wen et al.^45^); brain fog: ROR = 6.57 (95% CI 3.28-13.16) (Wen et al.^45^); euphoric mood: ROR = 6.01 (95% CI 2.25-16.04) (Wen et al.^45^) | Visual hallucination: ROR = 44.55 (95% CI 9.96-199.15) (Battini et al.^25^); personality change: ROR = 25.05 (95% CI 5.60-111.98) (Battini et al.^25^); hemiparesis: ROR = 5.47 (95% CI 1.76-16.98) (Cao et al.^29^); mental impairment: ROR = 4.97 (95% CI 1.87-13.27) (Cao et al.^29^); dysstasia: ROR = 4.74 (95% CI 1.97-11.4) (Cao et al.^29^); sedation: ROR = 4.72 (95% CI 1.77-12.59) (Cao et al.^29^); feeling drunk: ROR (95% CI lower 13.8) (Liang et al.^31^); euphoric mood: ROR (95% CI lower 11.2) (Liang et al.^31^); anxiety: disproportionality signal found (Song et al.^43^); insomnia: SDR found (Song et al.^43^); seizure: SDR found (Song et al.^43^) |  |
|  | **Gastrointestinal Effects (other than constipation)** | Oral discomfort: ROR = 5.86 (95% CI 1.94-17.67) (Battini et al.^25^); stomatitis: ROR = 4.39 (95% CI 1.27-15.19) (Battini et al.^25^); gastritis: ROR = 3.14 (95% CI 1.10-8.95) (Battini et al.^25^); dry mouth: ROR = 2.59 (95% CI 1.34-4.99) (Battini et al.^25^); vomiting projectile: SDR found (Hu et al.^30^); SDR found for abdominal discomfort: a higher likelihood was reported in patients aged 18-65 years vs. >65 years: ROR = 2.98 (95% CI 1.08-8.25) (Hu et al.^30^); eructation: SDR found (Hu et al.^30^); vomiting projectile: ROR (95% CI lower 6.31) (Liang et al.^31^), dyspepsia: ROR (95% CI lower 5.33) (Liang et al.^31^); vomiting projectile: ROR = 6.94 (95% CI 2.23-21.57) (Pan and Lin^33^); dyspepsia: ROR = 5.25 (95% CI 4.24-6.5) (Pan and Lin^33^); abdominal pain upper: ROR = 4.38 (95% CI 3.74-5.13) (Pan and Lin^33^); eructation: ROR = 3.73 (95% CI 2.07-6.75) (Pan and Lin^33^); dyspepsia: ROR = 5.25 (95% CI 4.24-6.5) (Pan and Lin^33^); vomiting: ROR = 3.25 (95% CI 2.69-3.91) (Lee et al.^40^); abdominal pain upper: SDR found (Song et al.^43^); dyspepsia: SDR found (Song et al.^43^); abdominal discomfort: SDR found (Song et al.^43^) | Impaired gastric emptying: ROR (95% CI lower 6.71) (Liang et al.^31^); gastrointestinal motility disorder: ROR (95% CI lower 5.16) (Liang et al.^31^); paresthesia oral: ROR = 4.58 (95% CI 2.05-10.2) (Zhang et al.^36^); impaired gastric emptying: ROR = 6.01 (95% CI 2.5-14.48) (Zhang et al.^36^); gastrointestinal motility disorder: ROR = 6.16 (95% CI 1.98-19.14) (Zhang et al.^36^); abdominal pain upper: SDR found (Song et al.^43^); abdominal discomfort: SDR found (Song et al.^43^); paraesthesia oral: ROR = 5.03 (95% CI 2.26-11.22) (Wen et al.^45^); impaired gastric emptying: ROR = 6.01 (95% CI 2.5-14.48) (Wen et al.^45^); gastrointestinal motility disorder: ROR = 6.77 (95% CI 2.18-21.04) (Wen et al.^45^) | Dysphagia: ROR = 5.01 (95% CI 1.49-16.87) (Battini et al.^25^); paresthesia oral: ROR = 12.71 (95% CI 5.7-28.33) (Cao et al.^29^); Paraesthesia oral: ROR (95% CI lower 10.08) (Liang et al.^31^); abdominal pain upper: SDR found (Song et al.^43^); abdominal discomfort: SDR found (Song et al.^43^) | Abdominal pain upper: SDR found (Song et al.^43^); abdominal discomfort: SDR found (Song et al.^43^) |
| **Special Populations** | **Safety in Pregnancy** | No SDR found (Liang et al.^31^); exposure during pregnancy: ROR = 4.33 (95% CI 3.33-5.64) (Pan and Lin^33^) | No SDR found (Liang et al.^31^); pregnancy: ROR = 8.76 (95% CI 4.55-16.87) (Zhang et al.^36^); pregnancy: ROR = 9.75 (95% CI 5.06-18.78) (Wen et al.^45^) | Habitual abortion: ROR (95% CI lower) = 5,982.5 (2,928.89) (Liang et al.^31^); no SDR found (Cao et al.^29^) |  |
|  |  | No SDR found. Analysis was conducted for the gepant class as a whole, which showed no disproportionate reporting of pregnancy-related events vs. triptans: ROR = 0.83 (95% CI 0.61-1.13). The study did not provide data stratified by individual drug (Noseda et al.^32^). | | | |

AEs: Adverse Events; AESIs: Adverse Events of Special Interest; CI: Confidence Interval; CV: Cardiovascular; ROR: Reporting Odds Ratio; SDR: Signal of Disproportionate Reporting. Note: The magnitude of disproportionality metrics (e.g. ROR) presented in this table varies depending on the comparator group (e.g., all other drugs vs. specific migraine treatments) and statistical method used in the primary studies. Values should be interpreted within the context of the individual study design and not directly compared across different analyses. The term 'SDR found' is used when a source article confirms a statistically significant disproportionality signal but does not report the specific risk metric value (e.g., ROR) and/or its 95% Confidence Interval. The term 'No SDR found' indicates that an analysis was conducted, but the results were not statistically significant. The term 'No reports found' is used when a study explicitly states that no adverse event reports were found for that specific drug-event combination.
